# Haematopoietic stem and progenitor cell heterogeneity is inherited from the embryonic endothelium

**DOI:** 10.1038/s41556-023-01187-9

**Published:** 2023-07-17

**Authors:** Joey J. Ghersi, Gabriel Baldissera, Jared Hintzen, Stephanie A. Luff, Siyuan Cheng, Ivan Fan Xia, Christopher M. Sturgeon, Stefania Nicoli

**Affiliations:** 1 Yale Cardiovascular Research Center, Department of Internal Medicine, Section of Cardiology, Yale University School of Medicine, New Haven, CT USA; 2 Department of Genetics, Yale University School of Medicine, New Haven, CT USA; 3 Vascular Biology & Therapeutics Program, Yale University School of Medicine, New Haven, CT USA; 4 Black Family Stem Cell Institute, Icahn School of Medicine at Mount Sinai, New York, NY USA; 5 Center for Advancement of Blood Cancer Therapies, Icahn School of Medicine at Mount Sinai, New York, NY USA; 6 Department of Cell, Developmental and Regenerative Biology, Icahn School of Medicine at Mount Sinai, New York, NY USA

**Keywords:** Haematopoietic stem cells, Haematopoiesis

## Abstract

Definitive haematopoietic stem and progenitor cells (HSPCs) generate erythroid, lymphoid and myeloid lineages. HSPCs are produced in the embryo via transdifferentiation of haemogenic endothelial cells in the aorta–gonad–mesonephros (AGM). HSPCs in the AGM are heterogeneous in differentiation and proliferative output, but how these intrinsic differences are acquired remains unanswered. Here we discovered that loss of microRNA (miR)-128 in zebrafish leads to an expansion of HSPCs in the AGM with different cell cycle states and a skew towards erythroid and lymphoid progenitors. Manipulating miR-128 in differentiating haemogenic endothelial cells, before their transition to HSPCs, recapitulated the lineage skewing in both zebrafish and human pluripotent stem cells. miR-128 promotes Wnt and Notch signalling in the AGM via post-transcriptional repression of the Wnt inhibitor *csnk1a1* and the Notch ligand *jag1b*. De-repression of *cskn1a1* resulted in replicative and erythroid-biased HSPCs, whereas de-repression of *jag1b* resulted in G2/M and lymphoid-biased HSPCs with long-term consequence on the respective blood lineages. We propose that HSPC heterogeneity arises in the AGM endothelium and is programmed in part by Wnt and Notch signalling.

## Abstract (web summary)

Ghersi et al. report that haematopoietic stem and progenitor cell heterogeneity is established on the haemogenic endothelium level and is, at least in part, regulated by microRNA-128-mediated modulation of Wnt and Notch signalling.

## Main

In the classical model of haematopoiesis, a homogeneous pool of haematopoietic stem cells (HSCs) proliferates while generating multipotent progenitors that, by following a stepwise restriction of lineage potential, generate all mature blood and immune cells^1^. This model has been challenged by evidence for molecular and functional heterogeneity within the HSC pool. HSC transplantation, barcoding and fate mapping experiments showed that only a few HSCs can produce all blood cells, while the majority of HSC differentiation is restricted or imbalanced to a few lineages^2–7^. Furthermore, HSCs differ in their proliferative capacity, which influences self-renewal kinetics^8,9^, with some HSCs generating specific blood cells without undergoing cell division^10^. Single-cell sequencing analysis confirmed that adult HSCs are a heterogeneous mixture of haematopoietic stem and progenitor cells (HSPCs) having different cell cycle status, transcriptional lineage priming and blood lineage outputs^11,12^. How HSPCs acquire these intrinsic phenotypic differences is currently unknown. This lack of knowledge is critical to understand how to regulate the HSPC production in vivo, as well as ex vivo where HSPC heterogeneity influences the success of autologous HSC transplantation in clinic^13,14^.

Cell tracing experiments of arterial haematopoietic clusters that form during embryonic development revealed the production of multiple HSPC clones; these clones migrate into the definitive haematopoietic organs, where they display long-term engraftment lineage biases (for example, lymphoid or myeloid) in juvenile and adult stages^2,5,15,16^. HSPC heterogeneity is therefore observed in the embryonic aorta–gonad–mesonephros (AGM) where nascent HSPCs (nHSPCs) are made from the transdifferentiation of arterial endothelial cells (ECs) specified into progenitor-like haemogenic EC (hemECs), before endothelial-to-haematopoietic transition (EHT)^17–20^. Whether and how ECs or hemECs contribute to long-term nHSPC phenotypes is unknown.

In this Article, using single-cell RNA sequencing (scRNA-seq) and phenotypic analysis of AGM ECs in nHSPC lineage priming models in vivo and in vitro, we discovered a previously unappreciated and unexpected mechanism in the endothelium that regulates nHSPC heterogeneity before EHT.

## Results

### miR-128 regulates nHSPC and blood lineage production

**Fig. 1:**
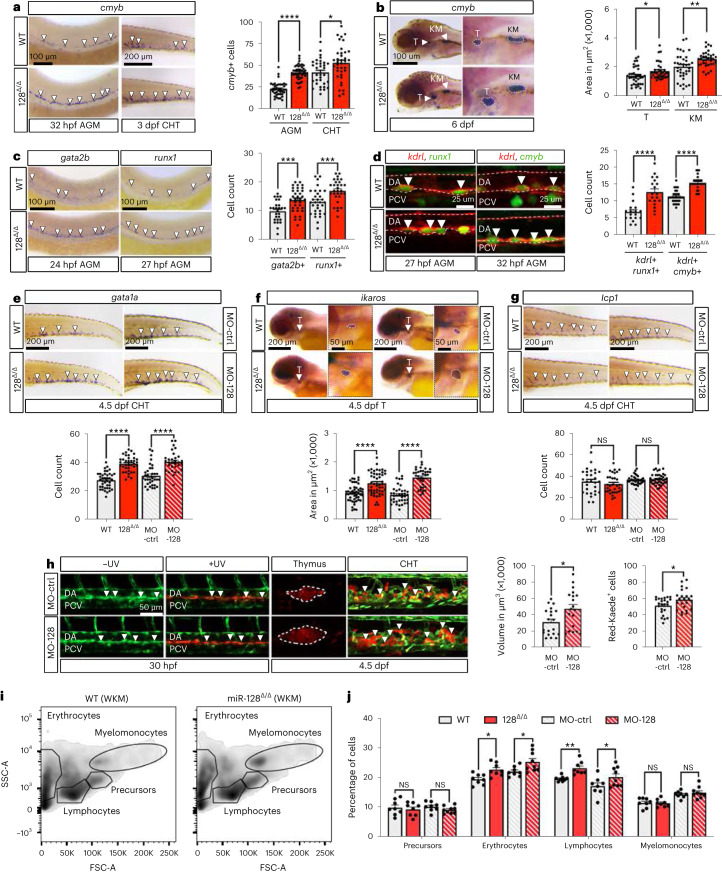
nHSPC development and blood lineages are altered in miR-128^Δ/Δ^. **a**, WISH against *cmyb* at 32 hpf and 3 dpf in WT or miR-128^Δ/Δ^ (128^Δ/Δ^) AGM (*n* = 39 (WT) and 42 (128^Δ/Δ^) embryos; *P* < 0.0001) and CHT (*n* = 33 (WT) and 48 (128^Δ/Δ^) embryos; *P* = 0.0151) (3 independent experiments, two-tailed Mann–Whitney test). **b**, WISH against *cmyb* at 6 dpf in WT (thymus *n* = 36 and KM *n* = 36 embryos; *P* = 0.0133) and 128^Δ/Δ^ (thymus *n* = 35 and KM *n* = 36 embryos; *P* = 0.0033; 3 independent experiments; two-tailed Mann–Whitney test). **c**, WISH against *gata2b* (*n* = 27 (WT) and 35 (128^Δ/Δ^) embryos; *P* = 0.0005) and *runx1* (n = 32 (WT) and 30 (128^Δ/Δ^) embryos; *P* = 0.0009; 3 independent experiments; two-tailed Mann–Whitney test). **d**, Confocal images of Tg(*kdrl:mCherry*^*s896*^*,cmyb:GFP*^*zf169*^*)* (*n* = 18 (WT) and 19 (128^Δ/Δ^) embryos; *P* < 0.0001) and Tg(*kdrl:mCherry*^*s896*^*,runx1:GFP*^*y509*^*)* (*n* = 29 (WT) and 31 (128^Δ/Δ^) embryos; *P* < 0.0001) AGM at 27 and 32 hpf, respectively. Quantification represents *runx1*+, *kdrl*+ (hemECs) and *cmyb*+, *kdrl*+ (nHSPCs) cells (3 independent experiments; two-tailed Mann–Whitney test). **e**–**g**, WISH of *gata1a* (*n* = 40 (WT), 37 (128^Δ/Δ^), 34 (MO-ctrl) and 31 (Mo-128) embryos; *P* < 0.0001) (**e**), *ikaros* (*n* = 48 (WT); 48 (128^Δ/Δ^), 37 (MO-ctrl) and 34 (Mo-128) embryos; *P* < 0.0001) (**f**) and *lcp1* (*n* = 29 (WT), 32 (128^Δ/Δ^), 36 (MO-ctrl), 38 (Mo-128) embryos; *P* = 0.0511 and 0.4257) (**g**) at 4.5 dpf with their quantification (3 independent experiments; two-tailed Mann–Whitney test). **h**, Confocal live imaging of Tg(*fli1a:Gal4*^*ubs4*^*,UAS:Kaede*^*rk8*^), ± UV (photoconversion) in the AGM. Quantification represents the red thymus area (MO-ctrl, 20; Mo-128, 19 embryos; *P* = 0.0151) and the number of red cells in the CHT (MO-ctrl, 19; Mo-128, 22 embryos; *P* = 0.0462; 3 independent experiments; two-tailed Mann–Whitney test). **i**, Flow cytometry analysis of 1-month-old dissected whole (W)KM WT, 128^Δ/Δ^. **j**, Quantification of cell population identified by flow cytometry (*n* = 8 (WT), 8 (128^Δ/Δ^), 8 (MO-ctrl) and 9 (MO-128) zebrafish; two-way ANOVA with multiple comparisons). All quantifications are represented with mean ± s.e.m. NS, not significant: *P* > 0.05, *P ≤ 0.05, **P ≤ 0.01, ***P ≤ 0.001, ****P ≤ 0.0001. Arrowheads indicate cells stained by WISH and IF and cells photoconverted in **h**. T, thymus; DA, dorsal aorta; PCV, posterior cardinal vein; SSC-A, side scatter A; FSC-A, forward scatter A. Source data

**Extended Data Fig. 1:**
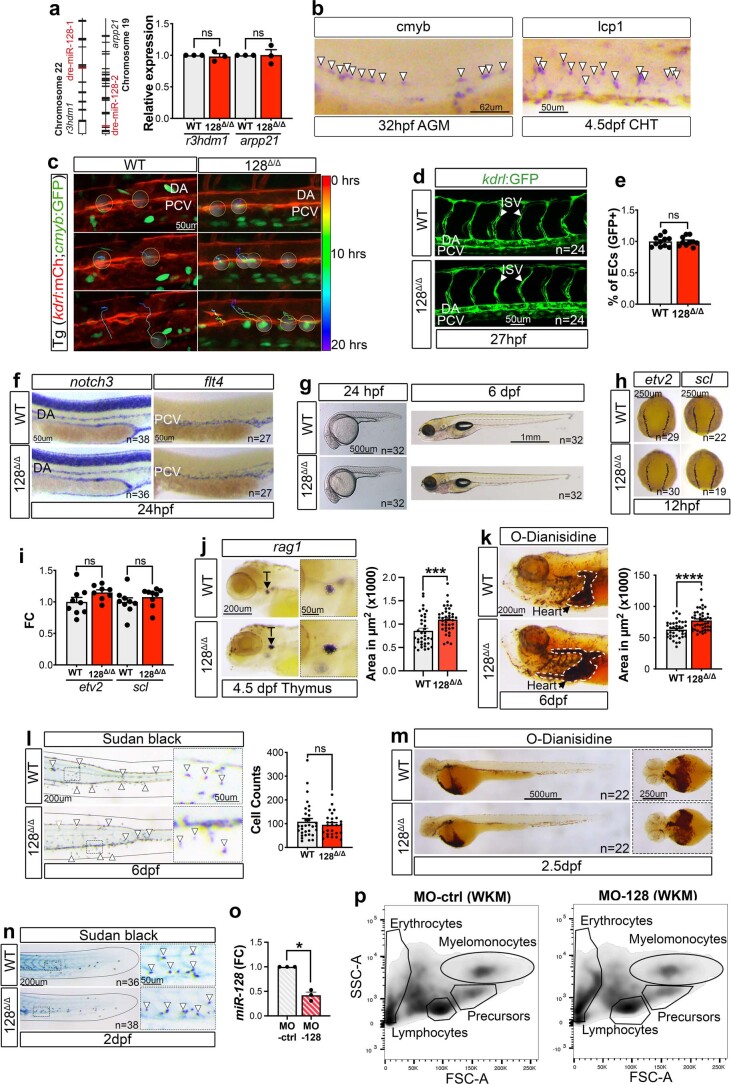
*miR-128*^*Δ/Δ*^*phenotype in vascular and hematopoietic development*. (**a**) Genomic location of *miR-128-1* and *miR-128-2* and qRT-PCR analysis of *r3hdm1* and *arpp21* (3 independent pools of ~10 embryos; Two-tailed Mann-Whitney test). (**b**) Representative images showing *cmyb* and *lcp1* cells. (**c**) Time-lapse with dragon-tail analysis of WT and 128^Δ/Δ^ Tg(*kdrl:mCherry*^*s896*^*,cmyb:GFP*^*zf169*^*)* from ~24 to 50 hpf. The color scale represents the time a cell to delaminate. (**d**) Confocal imaging of WT and 128^Δ/Δ^ embryos at 27 hpf. (**e**) Percentage of ECs over total number of cells in WT (n = 11 embryos) and 128^Δ/Δ^ (n = 10 embryos) at 26 hpf by flow cytometry (3 individual experiments, Two-tailed Mann-Whitney test). (**f**) WISH of WT and 128^Δ/Δ^ with *notch3* and *flt4*. (**g**) Images of WT and 128^Δ/Δ^ embryos. (**h**) WISH of *etv2* and *scl* at 12 hpf. (**i**) qRT-PCR analysis of *etv2* and *scl* (8 to 9 independent pools of ~10 embryos) at 12 hpf (Two-tailed Mann-Whitney test). (**j**) WISH of *rag1*+ cells at 4.5 dpf (n = WT, 36, 128^Δ/Δ^, 39 embryos; p-value = 0.001; 3 independent experiments, Mann-Whitney test). (**k**) O-dianisidine staining at 6 dpf (n = WT, 38, 128^Δ/Δ^, 44 embryos; p-value < 0.0001; 3 independent experiments, Mann-Whitney test). (**l**) Sudan black staining at 6 dpf (n = WT, 32, 128^Δ/Δ^, 27 embryos; 3 independent experiments, Two-tailed Mann-Whitney test). (**m**) O-dianisidine staining of WT and 128^Δ/Δ^ at ~2.5 dpf. (**n**) Sudan black staining of WT and 128^Δ/Δ^ at ~2 dpf. (**o**) qRT-PCR analysis of miR-128 normalized with U6 (n = MO-ctrl, 3, MO-128; p-value = 0.0224; 3 independent pools of ~10 embryos, Two-tailed one sample t and Wilcoxon test). (**p**) Flow cytometry population analysis of ~1-month-old dissected whole KM zebrafish injected with MO-control or MO-128. All quantifications are represented with mean ± SEM. ns: p > 0.05, *p ≤ 0.05. Abbreviations: aorta gonad mesonephros (AGM), dorsal aorta (DA), posterior cardinal vein (PCV), caudal hematopoietic tissue (CHT), thymus (T), intersegmental vessel (ISV) and (kidney marrow (KM).

The microRNA (miRNA) miR-128 is a highly conserved intronic miRNA that is enriched in embryonic ECs^21,22^ and is regulated in normal and malignant adult haematopoiesis^23–25^. We noticed that zebrafish embryos lacking the expression of both *miR-128-1* and *miR-128-2* (hereafter, miR-128^Δ/Δ^ (ref. ^22^)) displayed an increased number of cells expressing the nHSPC marker *cmyb* (Fig. 1a,b). The expression of *r3hdm1* and *arpp21*, miR-128 host genes, was unchanged in miR-128^Δ/Δ^ (Extended Data Fig. 1a), suggesting that miR-128 loss contributes to the increased nHSPC production. Relative to wild type (WT), miR-128^Δ/Δ^ displayed an increased number of nHSPCs in the embryonic AGM during EHT at 32 hours post fertilization (hpf) and in the secondary haematopoietic organ, the caudal haematopoietic tissue (CHT), the equivalent of the foetal liver in mammals, at 3 days post fertilization (dpf) (Fig. 1a and Extended Data Fig. 1b). The expansion of HSPCs was further noted at 6 dpf in the definitive haematopoietic organs, the thymus and the kidney marrow (KM), the equivalent of the bone marrow in mammals (Fig. 1b).

Intriguingly, *gata2b+* and *runx1+* hemECs, direct precursors of nHSPCs in the AGM, were also increased in miR-128^Δ/Δ^ (Fig. 1c). Live imaging of miR-128^Δ/Δ^ AGM in the transgenic endothelial-nHSPC reporter line *Tg(kdrl:mCherry*^*s896*^*;cmyb:GFP*^*zf169*^*)*^19^ and the hemEC reporter line *Tg(kdrl:mCherry*^*s896*^*;runx1:GFP*^*y509*^) (ref. ^26^) confirmed the expansion of nHSPCs and hemECs, respectively (Fig. 1d and Extended Data Fig. 1c). Notably, miR-128^Δ/Δ^ vascular development and morphogenesis was normal compared with WT (Extended Data Fig. 1d–i), supporting that nHSPC expansion is linked to an increase in EHT.

Next, we examined HSPC lineages in miR-128^Δ/Δ^. We found that erythroid (*gata1a*+) (ref. ^27^) and lymphoid (*ikaros*+) (ref. ^28^) progenitor cells were significantly expanded in the 4.5 dpf miR-128^Δ/Δ^ CHT and thymus compared with WT (Fig. 1e,f). Correspondingly, mature haemoglobin+ erythroid cells^29^ and *rag1*+ (ref. ^30^) B- and T-cell lymphopoietic tissues were also expanded in miR-128^Δ/Δ^ (Extended Data Fig. 1j,k). In contrast, the myeloid progenitors (*lcp1*+) (ref. ^31^) and mature cells such as Sudan black+ (ref. ^32^) neutrophils were unchanged in miR-128^Δ/Δ^ compared with WT (Fig. 1g and Extended Data Fig. 1b,l).

Given that primitive blood cells were unaffected by miR-128 loss (Extended Data Fig. 1m,n), we hypothesized that the excessive and biased progenitors observed in the secondary haematopoietic organs of miR-128^Δ/Δ^ are linked to the aberrant expansion of hemECs and nHSPCs during EHT in the AGM. To test this hypothesis, we induced transient downregulation of miR-128 in embryos by injecting 0.75 ng of morpholino, which was sufficient to recapitulate the biased expansion of erythroid and lymphoid progenitor cells that we detected in miR-128^Δ/Δ^ (Fig. 1e–g and Extended Data Fig. 1o). Moreover, we used the transgenic line Tg(*fli1a*:GAL4^ubs4^; *Tol2*-UAS:*Kaede*^*rk8*^), which drives vascular expression of Kaede, a fluorescent protein that undergoes irreversible photoconversion from green to red fluorescence upon exposure to ultraviolet (UV) light^33^. Photoconversion of Kaede during EHT at 30 hpf resulted in red fluorescent ventral aortic *Kaede+ fli1a+* cells that subsequently migrated to the thymus and CHT at 4.5 dpf (Fig. 1h). Notably, the thymic volume and number of red fluorescent cells in the CHT were both elevated in embryos treated with the miR-128 morpholino versus control morpholino (Fig. 1h), further suggesting that the excessive EHT in the AGM led to increased blood progenitors in secondary haematopoietic organs. To corroborate this finding in definitive haematopoietic organs, we grew both miR-128 morphants and miR-128^Δ/Δ^ to 1-month-old stage and analysed by flow cytometry the whole KM (WKM) where several distinct blood populations could be resolved by light-scatter characteristics^34^. Both miR-128^Δ/Δ^ and miR-128 morphants resulted in an increase of cell fractions relative to mature erythrocytes and lymphoid cells^34^ but not myelomonocytic cells or immature precursor cells (Fig. 1i,j and Extended Data Fig. 1p). Overall, our data indicate that miR-128 expression during the embryonic EHT is required to limit the production of nHSPCs and long-term erythroid and lymphoid lineages.

### miR-128 regulates nHSPC heterogeneity in the AGM

**Fig. 2:**
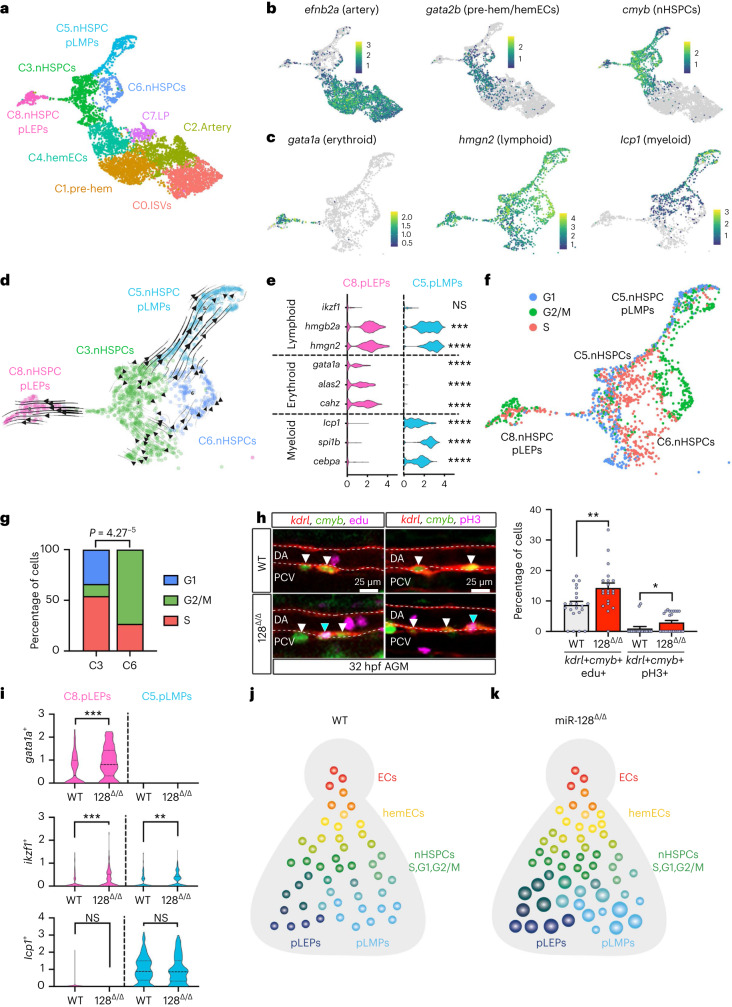
nHSPC heterogeneity is defined by cell cycle and lineage bias phenotypes and regulated by miR-128. **a**, UMAP of defined EHT cluster cells from *kdr*+ ECs in the tail of WT and 128^Δ/Δ^ at 26 hpf. **b**, UMAP representing *efnb2a*, *gata2b* and *cmyb* expression normalized with *Z*-score. **c**, UMAP of *gata1a*, *hmgn2* and *lcp1* expression normalized with *Z*-score, in nHSPC *cmyb*+ clusters (C3, C6, C8 and C5). **d**, RNA velocity trajectories in nHSPC clusters showing that C8.nHSPC pLEPs and C5.nHSPC pLMPs are terminal states of C3.nHSPCs and C6.nHSPCs. **e**, Violin plot of lymphoid (*ikzf1*, *P* > 0.9999; *hmgb2a*, *P* = 0.0001; *hmgn2*, *P* < 0.0001), erythroid (*gata1a*, *P* < 0.0001; *alas2*, *P* < 0.0001; *cahz*, *P* < 0.0001) and myeloid (*lcp1*, *P* < 0.0001; *spi1b*, *P* < 0.0001; *cebpa*, *P* < 0.0001) markers in WT cells of C8.nHPSC pLEPs and C5.nHSPC pLMPs. Statistics represent the comparison between C8.nHSPC pLEPs and C5.nHSPC pLMPs for each gene (ordinary one-way ANOVA). **f**, UMAP cell cycle analysis on nHSPC clusters. **g**, Quantification of S, G2/M and G1 phase in C3. and C6.nHSPCs. C3.nHSPCs cells are mainly in S phase and G1, while C6. nHSPCs in G2/M (two-tailed Student’s *t*-test with Bonferroni post-hoc correction). **h**, Confocal images of IF using anti-RFP, anti-GFP and EdU staining (*n* = 22 (WT) and 20 (128^Δ/Δ^) embryos; *P* = 0.0032) or anti-pH3 (*n* = 18 (WT) and 24 (128^Δ/Δ^) embryos; *P* = 0.0208) in Tg(*kdrl:mCherry*^*s896*^*,cmyb:GFP*^*zf169*^) AGM at 32 hpf. S phase and G2/M nHSPCs are increased (*kdrl*+, *cmyb*+, EdU+ or pH3+ blue arrowheads and *kdrl*+, *cmyb*+, EdU− or pH3− white arrowheads) in miR-128^Δ/Δ^ (three independent experiments; two-tailed Mann–Whitney test). **i**, Violin plot of *gata1a* (*P* = 0.0004)*, ikzf1* (*P* = 0.0006 and 0.0015) and *lcp1* (*P* = 0.4505 and 0.5383*)* expression in clusters C8.nHPSC pLEPs and C5.nHSPC pLMPs per genotype (Mann–Whitney test). **j**,**k**, Model of nHSPC heterogeneity acquired during EHT in the AGM at 26 hpf WT (**j**) and 128^Δ/Δ^ (**k**). 128^Δ/Δ^ nHSPC heterogeneity is biased towards S and G2/M nHSPCs (green circles), and erythroid and lymphoid primed nHSPCs (blue circles, bigger size represents increase gene expression but not number). Not signifcant (NS): *P* > 0.05. **P ≤ 0.01, ***P ≤ 0.001. LP, lymphatic progenitor; ISVs, intersegmental vessels; DA, dorsal aorta; PCV, posterior cardinal vein.

**Extended Data Fig. 2:**
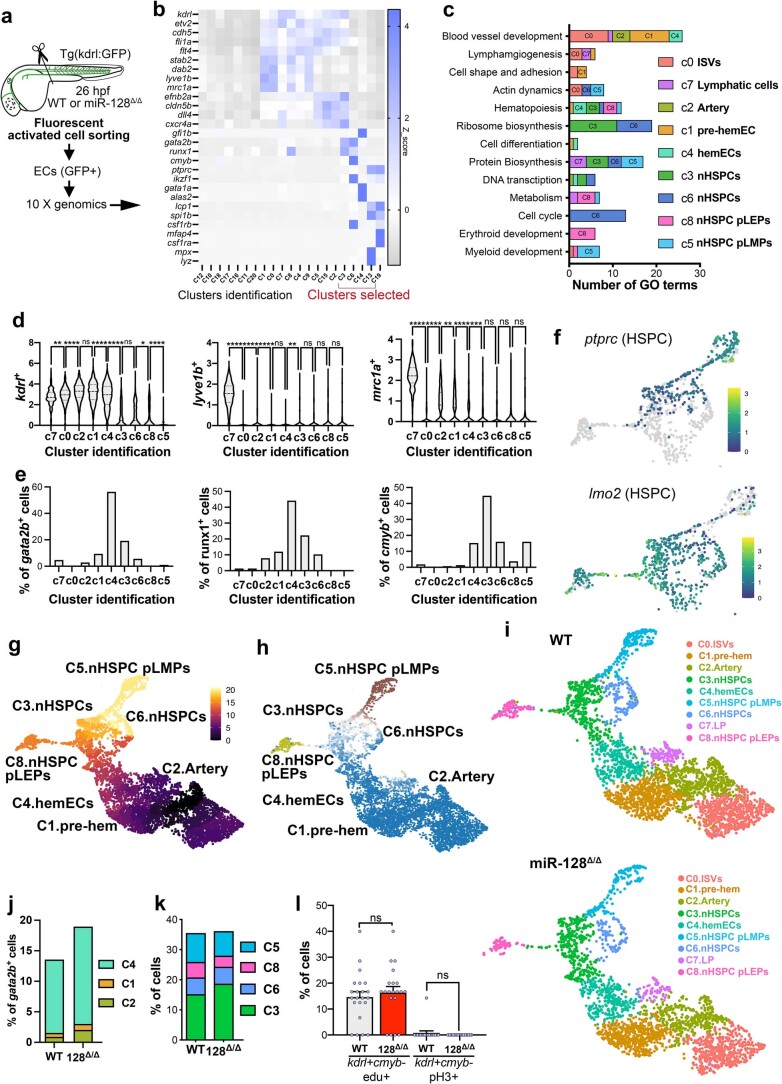
*Single cell RNA sequencing analysis of miR-128*^*Δ/Δ*^*EHT*. (**a**) Schematic representation of single cell RNA-sequencing (scRNAseq) strategy of *kdrl*+ tail endothelial cells (ECs) of WT and miR-128^Δ/Δ^ (128^Δ/Δ^) at 26 hpf isolated by FACS from Tg(*kdrl:GFP*^*zn1*^). (**b**) Heatmap showing the average gene expression of canonical vascular, hemogenic, blood stem cells and lineage gene markers in scRNAseq of 22,230 *kdrl*+ cells (WT + 128^Δ/Δ^). Clusters selected in red correspond to the ones used for the re-analysis of specific EHT cells. (**c**) Gene Ontology (GO) analysis of reanalyzed 6096 *kdrl* + EHT cells. (**d**) Violin plots of *kdrl*, *lyve1b* and *mrc1a* expression in each cluster of WT cells (ordinary one-way ANOVA with Tukey’s multiple comparison). (**e**) Percentage of cells expressing *gata2b*, *runx1* and *cmyb* in each cluster of WT cells. (**f**) UMAP of nHSPC clusters showing expression of defining HSPC markers, *ptprc* and *lmo2*. (**g,h**) Pseudotime analysis (**g**) and CellRank analysis (**h**) showing C8.nHSPC pLEPs and C5.nHSPC pLMPs are both terminal states of EHT cell clusters (Methods). (**i**) UMAP of WT and 128^Δ/Δ^ of EHT cluster *kdrl+* cells with assigned identification. (**j**) Percentage of cells expressing *gata2b* in pre-EHT (C2 and C1) and hemEC (C4) clusters in WT and 128^Δ/Δ^ *kdrl+* cells. (**k**) Percentage of cells in nHSPC clusters per genotype, showing the tendency of C3.nHSPCs to increase in 128^Δ/Δ^ compared to WT. (**l**) Quantification of ECs (*kdrl*+, *cmyb*^−^) edu+ (n = WT, 22; 128^Δ/Δ^, 20 embryos) or pH3+ (n = WT, 18; 128^Δ/Δ^, 24 embryos) counted in the same images as Fig. 2h (3 independent experiments, Two-tailed Mann-Whitney test). All Quantification are represented with mean ± SEM. ns: p > 0.05, *p ≤ 0.05, **p ≤ 0.01, *** p ≤ 0.001, **** p ≤ 0.0001. Abbreviations: primed lympho-erythroid progenitors (pLEPs), primed lympho-myeloid progenitors (pLMPs), lymphatic progenitor (LP) and intersegmental vessels (ISV).

To characterize the EHT in miR-128^Δ/Δ^ on a molecular level, we performed scRNA-seq of 22,230 *kdrl*+ ECs isolated from the tail of WT and miR-128^Δ/Δ^ 26 hpf (Extended Data Fig. 2a,b and Supplementary Table 1a). Of these cells, we focused on 6,096 cells expressing known vascular, arterial and haematopoietic markers, composed of nine different clusters (C) that represent the continuous progression of EHT in both WT and mutant cells (Extended Data Fig. 2c and Supplementary Table 1b). We identified tip cells (C0), two arterial cell clusters (C1 and C2) and one cluster of cells co-expressing arterial and lymphatic genes (C7) (Fig. 2a and Extended Data Fig. 2c,d). Compared with C2 and C7, C1 had a higher percentage of putative hemECs expressing *gata2b* and *runx1*, and thus was defined as a pre-haemogenic cluster (Fig. 2a,b, Extended Data Fig. 2d,e and Supplementary Table 1c,d). C4, adjacent to C1, was composed mostly of cells expressing a continuum of hemEC (*runx1* and *gata2b*) and nHSPCs (*cmyb*) markers, and therefore was defined as hemECs undergoing EHT (Fig. 2a,b and Extended Data Fig. 2e).

Cells emerging from the EHT C4 were grouped in four different nHSPC clusters having higher expression of *cmyb* as well as other known stem cell genes such as *lmo2* and *ptprc*^15^ and lower expression of the vascular marker *kdrl* (Fig. 2a,b and Extended Data Fig. 2d–f). As previously observed^15,19^, two of these nHSPC clusters showed high co-expression of genes priming lymphoid-erythroid progenitors (C8.nHSPC primed lympho-erythroid progenitors (pLEPs)) or priming lymphoid-myeloid progenitors (C5.nHSPC primed lympho-myeloid progenitors (pLMPs)) (Fig. 2c–e). In addition, we identified two groups of *cmyb*+ nHSPCs that mainly differed in the expression and distribution of cell cycle genes: C3.nHSPCs contained cells mainly in S phase and C6.nHSPCs contained cells mainly in G2/M phase (Fig. 2f,g and Supplementary Table 1e). RNA velocity^35^, pseudotime^36^ and CellRank^37^ analysis, which all predict computational trajectories of individual cells from scRNA-seq data, indicated that C5.nHSPC pLMPs and C8.nHSPC pLEPs were terminal states of C3. and C6.nHSPCs (Fig. 2d and Extended Data Fig. 2g,h). Thus, nHSPCs in early EHT have a continuum of phenotypes ranging from cell cycle to progenitor-biased states.

We then examined how miR-128 loss influences the composition of these clusters. Relative to WT, miR-128^Δ/Δ^ had an expanded population of *gata2b+* cells in the pre-EHT clusters (C2 and C1) and in C4 undergoing EHT, and an increase in the C3.nHSPCs post-EHT *cmyb*+ cluster (Extended Data Fig. 2i–k), consistent with our embryo analysis (Fig. 1a,c,d). To verify the cell cycle state of nHSPCs we visualized *cmyb*+, *kdrl+* cells in S phase and G2/M phase by the incorporation of 5-ethynyl-2′-deoxyuridine (EdU) and the staining of pH3, respectively. We observed an increase in S-phase and G2/M-phase nHPSCs in the AGM of miR-128^Δ/Δ^ versus WT (Fig. 2h and Extended Data Fig. 2l).

Interestingly, C6.nHSPCs, C5.nHSPC pLMPs and C8.nHSPC pLEPs were not elevated in the AGM of miR-128^Δ/Δ^ embryos relative to WT (Extended Data Fig. 2k). However, in miR-128^Δ/Δ^, both C5.nHSPC pLMPs and C8.nHSPC pLEPs showed elevated expression of the lymphoid marker *ikzf1*, and C8.nHSPC pLEPs showed elevated expression of the erythroid marker *gata1a*; the expression of the myeloid marker *lcp1* was unchanged in these clusters (Fig. 2i and Supplementary Table 1d). Overall, the analysis of the AGM cells of miR-128^Δ/Δ^ revealed that miR-128 regulates the EHT of S, G2/M nHSPCs as well as erythroid and lymphoid primed nHSPCs (Fig. 2j,k).

### Endothelial miR-128 regulates the production of biased nHSPCs

**Extended Data Fig. 3:**
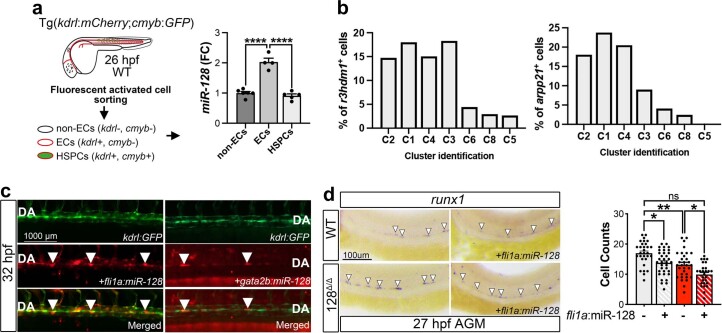
*miR-128 regulates nHSPC heterogeneity prior EHT in zebrafish*. (**a**) Schematic representation of FACS isolation of zebrafish cells from 26 hpf *Tg(kdrl:mCherry*^*s896*^*;cmyb:GFP*^*zf169*^*)* nHSPCs (*cmyb*+*, kdrl*+), endothelia cells (ECs) (*cmyb-,kdrl*+) and non-ECs (*cmyb-,kdrl-*). RT-qPCR analysis of *miR-128* (non-ECs, 5; ECs, 4; HSPCs, 5 independent pools of ~10 embryos) showing mature *miR-128* expression enriched in ECs vs HSPCs or non-ECs (ordinary one-way ANOVA with Tukey’s multiple comparison). (**b**) Percentage of cells in scRNA-seq clusters expressing *r3hdm1* and *arpp21* in which the intronic miR-128 genes 1 and 2 are hosted, respectively. (**c**) Confocal images of zebrafish tail at 32 hpf in WT Tg(*kdrl:GFP*^*zn1*^) injected at one cell stage with Tol2 *fli1a:miR-128-mCherry* and *gata2b:gal4* with Tol2 *UAS:miR-128-mCherry* (labeled as *gata2b:miiR-128)* (3 independent experiments). mCherry indicates successful *miR-128* expression in the respective cells (arrowheads). (**d**) WISH analysis of *runx1* (n = WT, 32; WT + *fli1a*, 27; 128^Δ/Δ^, 30; 128^Δ/Δ^ + *fli1a*, 35; embryos) at 27 hpf with and without Tol2 *fli1a:miR-128-mCherry* injection at one cell stage (3 independent experiments; ordinary one-way ANOVA with Tukey’s multiple comparison). All quantification are represented with mean ± SEM. ns: p > 0.05, *p ≤ 0.05, **p ≤ 0.01,**** p ≤ 0.0001. Abbreviations: aorta gonad mesonephros (AGM).

To investigate miR-128 expression during EHT, we performed fluorescence-activated cell sorting (FACS) of WT ECs, non-endothelial cells and nHSPCs using *Tg(kdrl:mCherry*^*s896*^*;cmyb:GFP*^*zf169*^*)* embryos at 26 hpf followed by quantitative reverse transcription polymerase chain reaction (qRT–PCR). Intriguingly, we found that miR-128 expression was higher in *kdrl+cmyb*− ECs than in *kdrl*+*cmyb*+ nHSPCs and *kdrl*−*cmyb*− non-endothelial cells (Extended Data Fig. 3a). Moreover, the expression of *r3hdm1* and *arpp21*, the genes hosting miR-128, were enriched in pre-EHT clusters and in clusters undergoing EHT in our scRNA-seq data (Extended Data Fig. 3b).

**Fig. 3:**
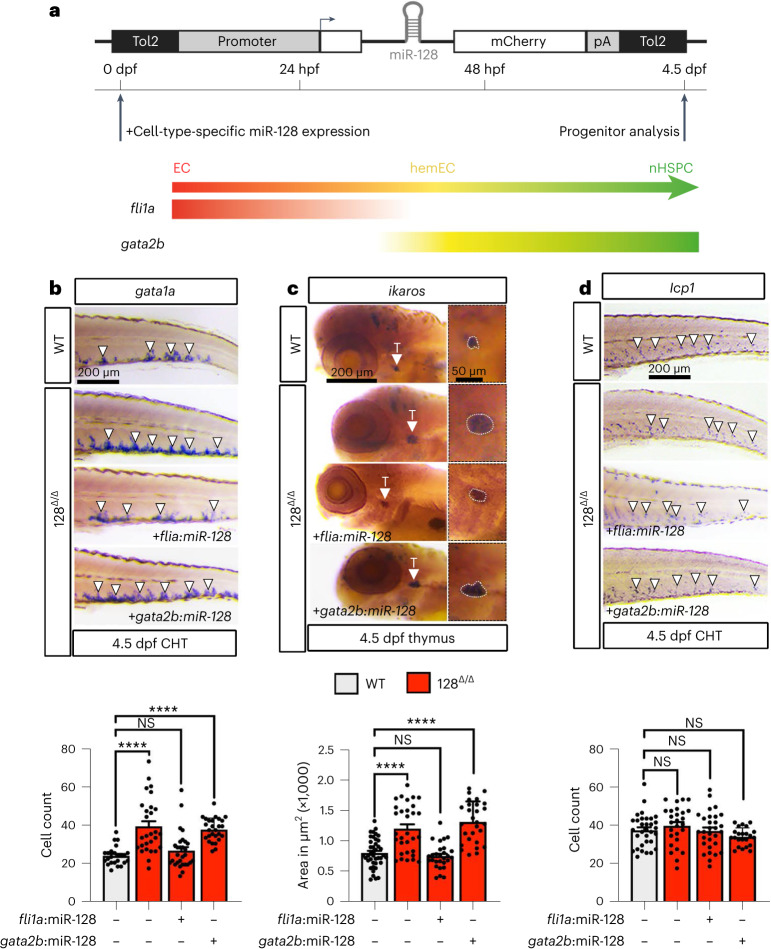
miR-128 function in ECs to regulate HSPC heterogeneity before EHT. **a**, Schematic representation of the transgene used to express WT miR-128 gene in zebrafish ECs (via *flia1*) or hemECs (via *gata2b*). For *gata2b* expression, we used the Tg(*gata2b*:Gal4^sd32^) line and created the *UAS:miR-128* plasmid, while for *fli1a* expression we created *fli1a*:*miR-128* plasmid. One-cell-state embryo was injected with tol2 mRNA and the indicated plasmids, and WISH was performed against *gata1a*, *ikzf1* and *lcp1* at 4.5 dpf, respectively. **b**–**d**, WISH of *gata1a* (*n* = 27 (WT), 28 (128^Δ/Δ^), 27 (128^Δ/Δ^ + *fli1a*), 29 (128^Δ/Δ^ + *gata2b*) embryos) (**b**) and *ikaros* (*n* = 41 (WT); 31 (128^Δ/Δ^), 28 (128^Δ/Δ^ + *fli1a*), 28 (128^Δ/Δ^ + *gata2b*) embryos) (**c**) and *lcp1* (*n* = 32 (WT), 27 (128^Δ/Δ^), 30 (128^Δ/Δ^ + *fli1a*), 23 (128^Δ/Δ^ + *gata2b*) embryos) (**d**) at 4.5 dpf and relative cells quantification as indicated. *fli1a* endothelial expression of *miR-128* WT gene rescues to WT level the increase of erythroid and lymphoid progenitors of miR-128^Δ/Δ^, while *gata2b* hemEC expression of miR-128 does not rescue the increase of erythroid and lymphoid progenitors (3 independent experiments, ordinary one-way ANOVA with Tukey’s multiple comparison). All quantifications are represented with mean ± s.e.m. NS, not significant; *P* > 0.05, ****P ≤ 0.0001.

Importantly, expressing a WT copy of miR-128 from the EC promoter *fli1a* (ref. ^38^) rescued the excessive number of *runx1+* hemECs/nHSPCs in the AGM of 27 hpf miR-128^Δ/Δ^, as well as the bias of the blood progenitors in the CHT and thymus at 4.5 dpf (Fig. 3a–d and Extended Data Fig. 3c,d). In contrast, expressing WT miR-128 from the haemogenic endothelium promoter *gata2b* from early stages, before *runx1* expression^39^, was unable to revert these miR-128^Δ/Δ^ phenotypes (Fig. 3a–d and Extended Data Fig. 3c). On the basis of these data, we suggest that miR-128 expression in ECs before haemogenic specification in the AGM is required for EHT and balanced blood progenitor production.

**Fig. 4:**
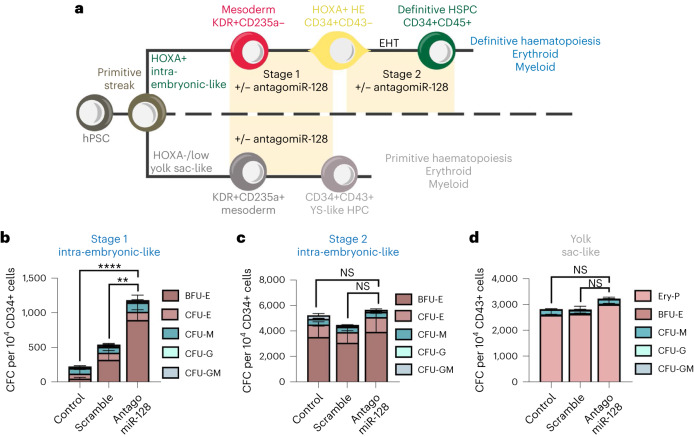
miR-128 function before EHT is conserved in hPSCs. **a**, Schematic of HSPC development in vitro using hPSCs and treated as indicated with antagomiR-128 or scramble miR as control at different cell stages. **b**,**c**, Colony-forming cell assay quantification of erythroid (BFU-E, CFU-E) and myeloid (CFU-M, CFU-G, CFU-GM) of *HOXA+* programme (definitive haematopoiesis) during stage 1 (**b**) or stage 2 (**c**). **d**, Colony-forming cell quantification of erythroid (Ery-P, BFU-E) and myeloid (CFU-M, CFU-G, CFU-GM) of *HOXA/low*− programme (primitive haematopoiesis) (three independent experiments). All Quantification are represented with mean ± s.e.m. NS, not significant; *P* > 0.05, **P ≤ 0.01, ****P ≤ 0.0001. HE, haemogenic endothelium; CFC, colony-forming cell; BFU-E, burst-forming units erythroid; CFU-E, colony-forming units of erythroid; CFU-G, colony-forming units of granulocyte; CFU-M, colony-forming units of myeloid; CFU-GM, colony-forming units of mixed granulocyte/myeloid.

**Extended Data Fig. 4:**
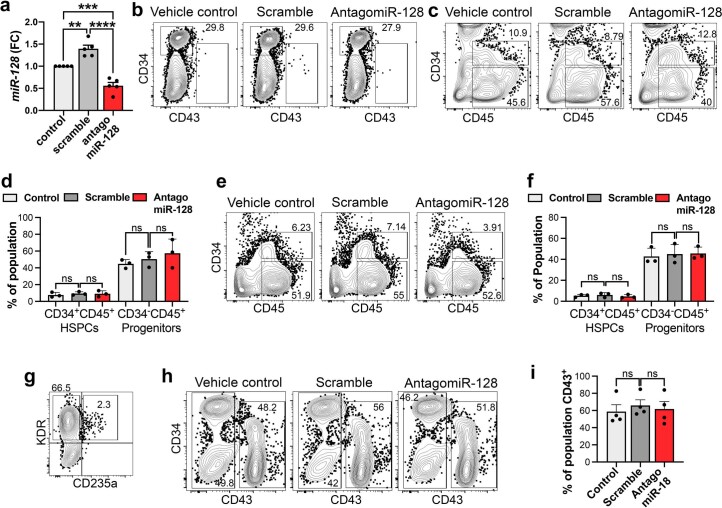
*miR-128 regulates nHSPC heterogeneity prior EHT in human pluripotent stem cells*. **a**) qRT-PCR analysis of mature *miR-128* in hemogenic endothelial cells from human pluripotent stem cell (hPSC) treated with scramble or antagomiR-128 at stage 1 (n = Control, 5; scramble, 5; antagomir-128, 5 independent experiments; ordinary one-way ANOVA with Tukey’s multiple comparison). (**b**) Representative flow cytometry analysis of CD34 and CD43 expression during differentiation of cells treated with vehicle control or scramble or antagomir-128 (Stage 1). (**c**) Representative flow cytometry analysis of cells expressing CD34 and CD45 during differentiation of hPSCs treated with vehicle control or scramble or antagomir-128 (Stage 1). (**d**) Quantification of cells CD34^+^CD45^+^ (HSPCs) (n = Control, 3; scramble, 3; antagomir-128, 3 independent experiments) and CD34^–^CD45^+^ (progenitors) (n = Control, 3; scramble, 3; antagomir-128, 3 independent experiments) (Ordinary one-way ANOVA with Tukey’s multiple comparison). (**e**) Representative flow cytometry analysis of cells expressing CD34 and CD45 during differentiation of hPSCs treated with vehicle control or scramble or antagomir-128 (Stage 2). (**f**) Quantification of cells CD34^+^CD45^+^ (HSPCs) (n = Control, 3; scramble, 3; antagomir-128, 3 independent experiments) and CD34^–^CD45^+^ (blood progenitors) (n = Control, 3; scramble, 3; antagomir-128, 3 independent experiments, ordinary one-way ANOVA with Tukey’s multiple comparison). (**g**) Representative flow cytometry analysis of cells expressing KDR and CD235a during hPSC differentiation. (**h**) Representative flow cytometry analysis of cells expressing CD34 and CD43 during hPSC differentiation treated with vehicle control or scramble or antagomiR-128. (**i**) Quantification of CD43^+^ cells (n = Control, 4; scramble, 4; antagomir-128, 4 independent experiments; ordinary one-way ANOVA with Tukey’s multiple comparison). All quantification are represented with mean ± SEM. ns: p > 0.05, **p ≤ 0.01, *** p ≤ 0.001,**** p ≤ 0.0001. Abbreviations: fold change (FC) and hemogenic endothelium (HE).

To corroborate this finding, we employed an in vitro system using human pluripotent stem cell (hPSC) differentiation to recapitulate the earliest stages of haematopoietic development via EHT (Fig. 4a). Briefly, we previously demonstrated that treating primitive streak-like cells with the Wnt activator CHIR99021 (GSK-3 inhibitor) specifies a KDR+CD235a− mesodermal population, which in turn gives rise to a *HOXA*+CD34+CD43− population that harbours intra-embryonic-like hemECs (stage 1) (refs. ^40,41^). These cells, in turn, undergo EHT to form CD34+CD45+ definitive HSPCs (stage 2) (refs. ^40,42–44^). These HSPCs can be assessed for their ability to generate definitive erythroblasts and myeloid cells (Fig. 4a)^44^. With this hPSC model, we used an antagomir^45^ to reduce miR-128 expression at stage 1 or stage 2 of HSPC differentiation (Extended Data Fig. 4a). Antagomir treatment during stage 1, the specification of *HOXA+* hemECs from mesoderm, resulted in an overall two- to five-fold increase in definitive erythroid, but not myeloid, output (Fig. 4b and Extended Data Fig. 4b–d). Notably, inhibition of miR-128 during differentiation of *HOXA+* hemEC into CD34+CD45+ HSPCs did not affect their lineage bias (Fig. 4c and Extended Data Fig. 4e,f), supporting that miR-128 expression before EHT is key to driving HSPC lineage phenotypes.

We also previously showed that treating primitive streak-like cells with the Wnt inhibitor IWP2 (PORCN inhibitor) and ACTIVIN A specifies a KDR+CD235a+ mesodermal population, which in turn gives rise to CD34+CD43+*HOXA*− yolk sac-like haematopoietic progenitor cells that can be assessed for their ability to give rise to primitive erythroblasts and myeloid cells (Fig. 4a)^43^. hPSCs treated with or without antagomir, during the differentiation of KDR+CD235a+, showed no changes in overall haematopoietic output (Fig. 4d and Extended Data Fig. 4g–i), suggesting that miR-128 does not play a role in primitive haematopoiesis, consistent with our observations in zebrafish (Extended Data Fig. 1m,n).

Overall, these data suggest that miR-128 functions in ECs, before EHT, to limit the generation of progenitor-biased nHSPCs.

### nHSPC heterogeneity is modulated by miR-128 via Wnt and Notch

**Extended Data Fig. 5:**
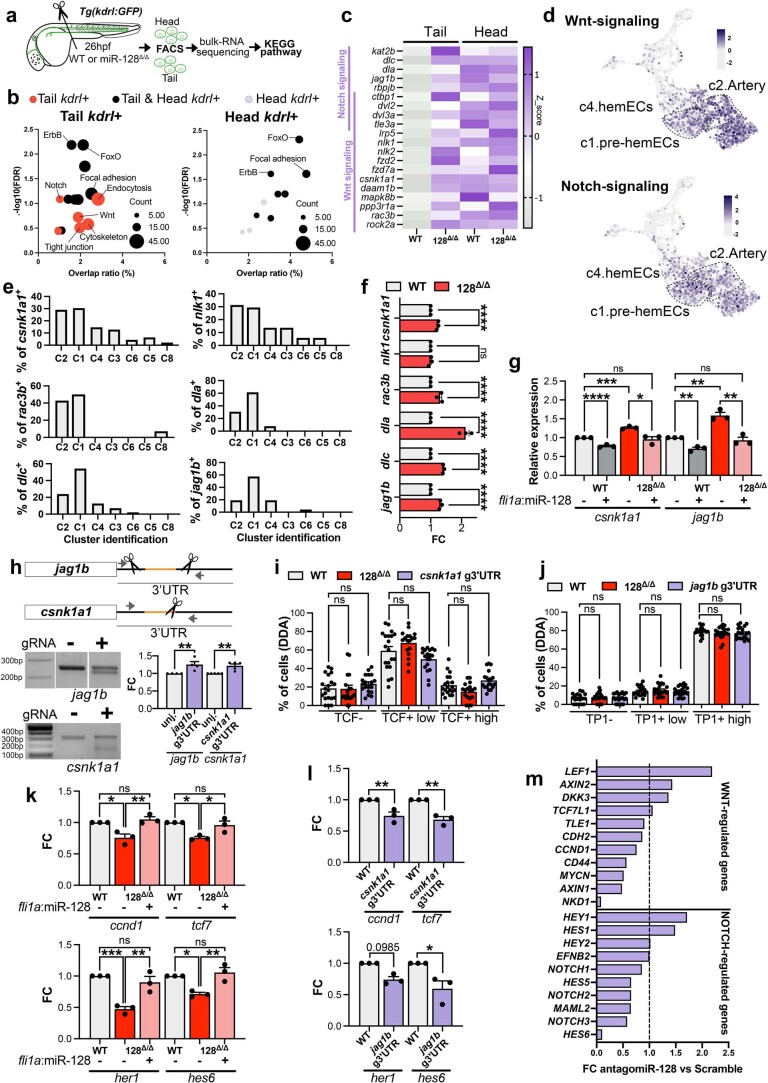
*miR-128 targets csnk1a and jag1b prior EHT to regulate Wnt and Notch signaling respectively*. (**a**) Bulk RNA sequencing strategy. (**b**) KEGG analysis on miR-128 target genes. (**c**) Heatmap of bulk RNA sequencing in WT and 128^Δ/Δ^. (**d**) UMAP of Wnt- and Notch-signaling signature in WT cells. (**e**) Percentage of cells expressing *csnk1a1*, *nlk1*, *rac3b*, *dla*, *dlc* and *jag1b* in scRNA-seq WT EHT clusters. (**f**) qRT-PCR at 26 hpf (n = 3 independent experiments for all genes and conditions, two-way ANOVA). (**g**) qRT-PCR of *csnk1a1* and *jag1b* at 26 hpf (3 independent pools of ~10 embryos, Ordinary one-way ANOVA with Tukey’s multiple comparison). (**h**) CRISPR/Cas9 design in miR-128 binding site (orange). Agarose gel of *csnk1a1* and *jag1b* 3′UTR region. qRT-PCR of *csnk1a1* and *jag1b* at 24 hpf (*jag1b* expression, n = WT, 3; *jag1b* g3′UTR, 3; *csnk1a1* expression; WT, 4; *csnk1a1* g3′UTR, 4 independent pools of ~5 embryos; Two-tailed Mann-Whitney test). (**i,j**) **(i)** Tg(*TCF:NLS-mCherry*^*ia5*^*,kdrl:eGFP*^*zn1*^) (n = WT, 20; 128^Δ/Δ^, 18; *csnk1a1* g3′UTR, 19 embryos) and **(j)** Tg(*TP1:eGFP*^*um14*^*,kdrl:mCherry*^*s896*^) (n = WT, 18; 128^Δ/Δ^, 20; *jag1b* g3′UTR, 21 embryos; 3 independent experiments; ordinary one-way ANOVA with Tukey’s multiple comparison). (**k**) qRT-PCR analysis of Wnt (*ccnd1* and *tcf7*) or Notch (*her1* and *hes6*) responsive genes in 24hpf WT and 128^Δ/Δ^ (For all conditions, n = 3 independent pools of ~10 embryos for all genes; ordinary one-way ANOVA). (**l**) qRT-PCR analysis of Wnt (*ccnd1* and *tcf7*) or Notch (*her1* and *hes6*) responsive genes in 24 hpf WT, *csnk1a1* g3′UTR and *jag1b* g3′UTR (For all conditions, n = 3 independent pools of ~10 embryos for all genes; ordinary one-way ANOVA). (**m**) Bar graph representing WNT- and NOTCH-regulated genes by bulk RNA-sequencing of HOXA + CD34 + CD43- cells following scramble or antagomiR-128 treatment during stage 1. All quantification are represented with mean ± SEM. ns: p > 0.05, *p ≤ 0.05, **p ≤ 0.01, *** p ≤ 0.001, **** p ≤ 0.0001. Abbreviations: T7 endonuclease (T7EI), KEGG (Kyoto Encyclopedia of Genes and Genomes).

To discern how miR-128 regulates nHSPC diversity in the AGM endothelium, we compared the transcriptomes of 26 hpf *kdrl*+ ECs from the tail of miR-128^Δ/Δ^ and WT embryos (Extended Data Fig. 5a). We used *kdrl*+ cells from heads as controls for specificity. Multiple signalling components were upregulated specifically in miR-128^Δ/Δ^ tail *kdrl*+ cells (Extended Data Fig. 5a,b and Supplementary Table 2a,b). As a proof-of-principle we focused on miR-128-mediated inhibition of Notch and canonical Wnt signalling genes because these are key pathways of in vivo and ex vivo HSPC production via EHT^43,46–48^ (Extended Data Fig. 5c and Supplementary Table 2c). Furthermore, these messenger RNAs were expressed predominantly in *kdrl+* pre-EHT cells (mainly arterial cells in C2 and C1) where miR-128 is functional, and were de-repressed in miR-128^Δ/Δ^ tails at 24 hpf, consistent with the loss of post-transcriptional inhibition (Extended Data Fig. 5d–f and Supplementary Table 1c).

Among these candidates we identified the negative regulator of Wnt signalling, casein kinase 1α (*csnk1a*) (ref. ^49^), which is not known to play a role in EHT, and the Notch ligand jagged 1b (*jag1b*), which plays a role in haemogenic cell specification^50–53^. Importantly, both *csnk1a1* and *jag1b* were de-repressed in miR-128^Δ/Δ^, and their levels were restored to normal after miR-128 expression in ECs (Extended Data Fig. 5g).

**Fig. 5:**
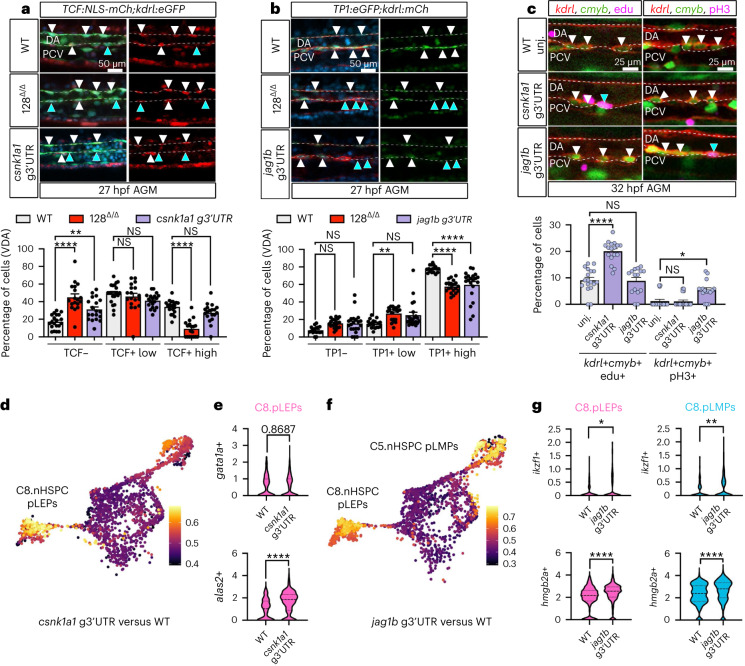
miR-128 regulation of Notch (via *jag1b*) and Wnt (via *csnk1a1*) signalling in the EHT control nHSPC heterogeneity. **a**,**b**, Confocal lateral view of IF zebrafish tail at 27 hpf of Tg(*TCF:NLS-mCherry*^*ia5*^*,kdrl:eGFP*^*zn1*^) (*n* = 20 (WT), 18 (128^Δ/Δ^) and 19 (*csnk1a1* g3′UTR) embryos) (**a**) and Tg(*TP1:eGFP*^*um14*^*,kdrl:mCherry*^*s896*^) (*n* = 18 (WT), 20 (128^Δ/Δ^) and 21 (*jag1b* g3′UTR) embryos) (**b**), Wnt and Notch reported lines, respectively. Quantification of Wnt and Notch *kdrl*+ cells based on reporter intensity. Arrowheads represent *kdrl*+, TCF+ or TP1+ high (white) or low/negative (blue) cells. Cell quantification is reported in the ventral floor of the dorsal aorta (VDA) (three independent experiments; ordinary one-way ANOVA with Tukey’s multiple comparison). **c**, Confocal images of Tg(*kdrl:mCherry*^*s896*^*,cmyb:GFP*^*zf169*^*)* WT and g3′UTR mutants AGM at 32 hpf. Replicative nHSPCs are increased (*kdrl*^+^, *cmyb*^+^, EdU+, blue arrowhead) (*n* = 19 (WT), 17 (*csnk1a1* g3′UTR) and 15 (*jag1b* g3′UTR) embryos) in the *csnk1a1* g3′UTR while unchanged in *jag1b* g3′UTR. G2/M nHSPCs (*kdrl*^+^, *cmyb*^+^, pH3+, blue arrowhead) (*n* = 17 (WT), 19 (*csnk1a1* g3′UTR), 16 (*jag1b* g3′UTR) embryos) are increased in the *jag1b* g3′UTR while unchanged in *csnk1a1* g3′UTR (3 independent experiments; ordinary one-way ANOVA with Tukey’s multiple comparison). **d**, scRNA-seq analysis of *kdrl+* cells experimental perturbation among genotype using MELD (Methods) assessed by the comparison between *csnk1a1* g3′UTR versus WT, showing C8 as the most perturbed cluster. **e**, Violin plot of erythroid markers (*gata1a* and *alas2*, *P* < 0.0001) within C8.nHSPC pLEPs (Mann–Whitney test). **f**, MELD assessed by the comparison between *jag1b* g3′UTR versus WT, showing C8 and C5 as the most perturbed clusters (Methods). **g**, Violin plot of lymphoid markers (*ikzf1*, *P* = 0.0315 (C8) and 0.0099 (C5) and *hmgb2a*, *P* < 0.0001) within C8.nHSPC pLEPs and C5.nHSPC pLMPs (two-tailed Mann–Whitney test). All quantifications are represented with mean ± s.e.m. NS, not significant; *P* > 0.05, *P ≤ 0.05, **P ≤ 0.01, ****P ≤ 0.0001. DA, dorsal aorta; PCV, posterior cardinal vein.

To disrupt miR-128-mediated regulation of these targets, we used clustered regularly interspaced short palindromic repeats (CRISPR)/Cas9 to mutate miR-128 binding sites in the *csnk1a1* or *jag1b* genomic (g) 3′ untranslated regions (UTRs) (Extended Data Fig. 5h). These genetic perturbations led to de-repression of the associated transcripts at 24 hpf (Extended Data Fig. 5h), consistent with loss of miR-128-mediated inhibition. To determine how *csnk1a1* or *jag1b* impact signalling pathways, we introduced these mutations into the *Tg(TCF:nls-mCherry*^*ia5*^*)* Wnt and *Tg(TP1:eGFP*^*um14*^*)* Notch reporter lines, respectively. We found, in the ventral floor of the dorsal aorta (the AGM), that *csnk1a1* g3′UTR mutants showed an increase in cells that lack expression of the Wnt reporter (Fig. 5a), and *jag1b* g3′UTR mutants showed a decrease in cells with high expression of the Notch reporter (Fig. 5b). The miR-128^Δ/Δ^ presented both of these phenotypes (Fig. 5a,b). In contrast, *kdrl*+ cells in the dorsal floor of the dorsal aorta, which does not undergo EHT, had similar Wnt and Notch activity in all the genotypes (Extended Data Fig. 5i,j). Additionally, conserved Notch and Wnt-signalling targets had reduced expression in miR-128^Δ/Δ^ and *csnk1a1* or *jag1b* g3′UTR mutants (Extended Data Fig. 5k,l), and in the aorta-like CD34+ cells derived from the hPSCs treated with the miR-128 antagomir during stage 1 (Extended Data Fig. 5m and Supplementary Table 3). Overall, these data suggest that post-transcriptional repression of *csnk1a1* and *jag1b* by miR-128 sustains Wnt and Notch signalling, respectively, before EHT.

**Fig. 6:**
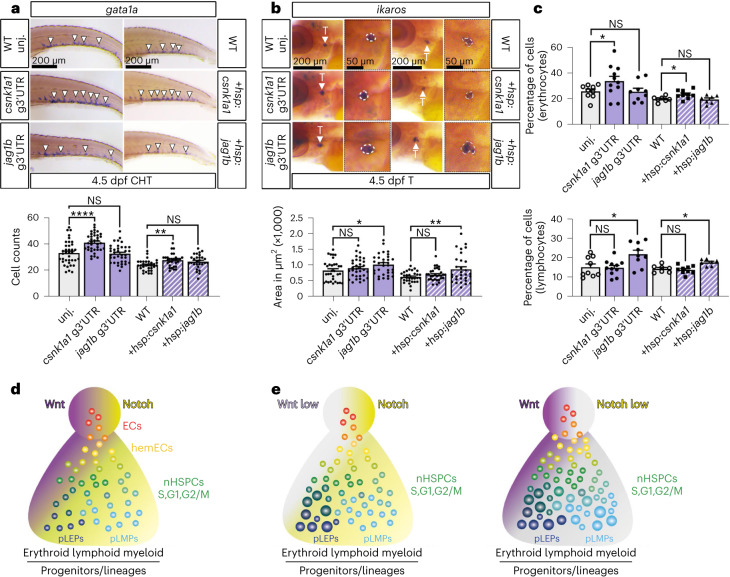
miR-128 regulation of jag1b and csnk1a1 in the embryonic EHT control long-term blood lineages. **a**,**b**, Quantification of erythroid (**a**) and lymphoid (**b**) progenitors by WISH against *gata1a* (*n* = 37 (WT), 37 (*csnk1a1* g3′UTR), 37 (*jag1b* g3′UTR), 31 (WT), 30 (*hsp:csnk1a1*) and 28 (*hsp:jag1b*) embryos) and *ikaros* (*n* = 32 (WT), 34 (*csnk1a1* g3′UTR), 32 (*jag1b* g3′UTR), 31 (WT), 31 (*hsp:csnk1a1*) and 30 (*hsp:jag1b*) embryos), respectively, at 4.5 dpf CHT or thymus. WT and *hsp:csnk1a1* and *hsp:jag1b* were heat shocked at 24 hpf. *csnk1a1* de-repression or transient gain-of-function regulate erythroid progenitor formation, while *jag1b* manipulations regulate lymphoid progenitors (three independent experiments; ordinary one-way ANOVA with Tukey’s multiple comparison). **c**, Quantification of blood cell population identified by flow cytometry of ~2-month-old WKM dissected from samples treated as in **h** and **i**. Erythrocyte fraction is increased in *csnk1a1* g3′UTR and *hsp:csnk1a1*, while lymphoid cells fraction is increased in *jag1b* g3′UTR and *hsp:jag1b* after heat shock at 24 hpf (*n* = 9 (WT), 11 (*csnk1a1* g3′UTR), 9 (*jag1b* g3′UTR), 7 (WT), 9 (*hsp:csnk1a1*) and 7 (*hsp:jag1b*) zebrafish; two-way ANOVA with multiple comparisons). **d**, Proposed model of nHSPC heterogeneity regulated by miR-128 regulation on Wnt and Notch signalling in vascular endothelia cells before EHT. Vascular signalling, like Wnt (purple) and Notch (yellow), limits the production of a heterogeneous pool of nHSPCs in the AGM. **e**, Diminishment of Wnt (light grey), like after de-repression or gain of function of *csnk1a1* in the EHT, results in an increase of replicative (S), and erythroid-biased nHSPCs in the AGM and relative erythroid progenitors and mature lineages. Diminishment of Notch (light grey), like after de-repression or gain of function of *jag1b* in the EHT, increases G2/M nHSPCs and lymphoid-biased nHSPCs in the AGM and relative lymphoid progenitors and mature cells. All quantifications are represented with mean ± s.e.m. NS, not significant; *P* > 0.05, *P ≤ 0.05, **P ≤ 0.01, ****P ≤ 0.0001. T, thymus.

**Extended Data Fig. 6:**
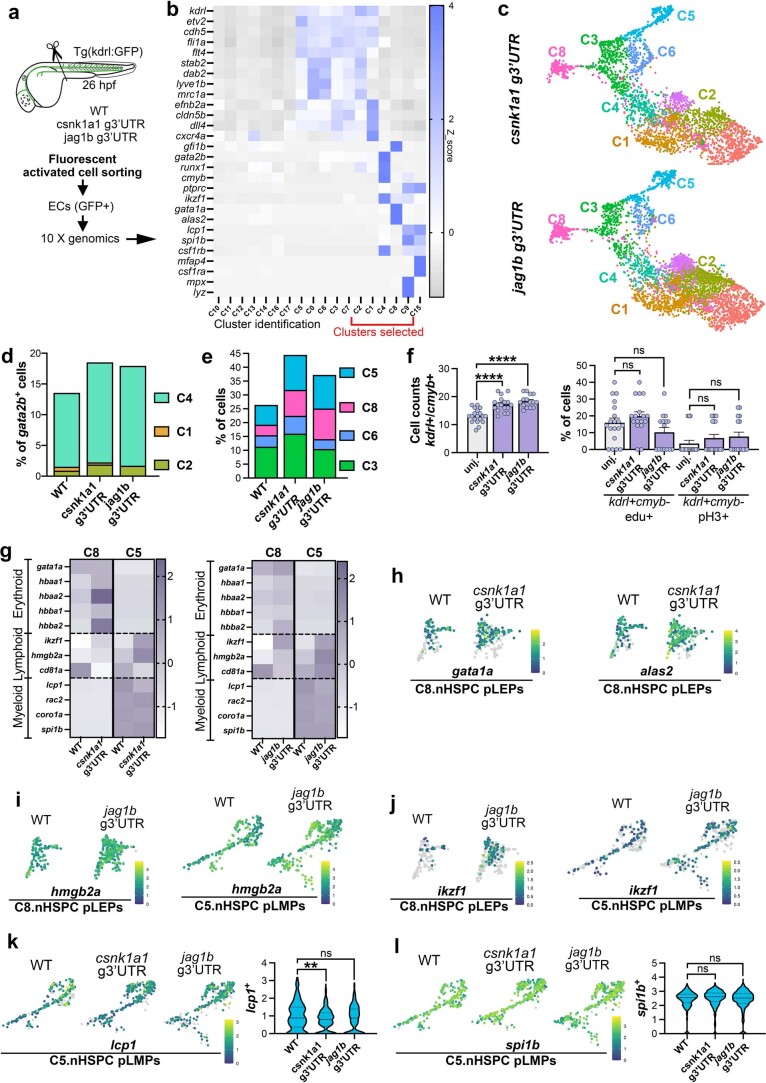
*miR-128 regulation of csnk1a1 and jag1b differentially guide nHSPC heterogeneity*. (**a**) scRNA seq strategy of WT, *csnk1a1* and *jag1b* g3′UTR Tg(*kdrl:*GFP^*zn1*^). (**b**) Heatmap of defining markers (20,600 *kdrl+* cells). (**c**) UMAP of 5597 *kdrl+* cells part of the clusters reanalyzed from EHT clusters in b. (**d**) Percentage of *gata2b*+ cells in pre-EHT clusters (C2, C1 and C4). (**e**) Percentage of cells in nHSPC clusters in all genotypes. (**f**) Quantification of nHSPCs (*cmyb*+*, kdrl*+) (n = WT, 19, *csnk1a1* g3′UTR, 17; *jag1b* g3′UTR, 16 embryos) at 32 hpf WT, *csnk1a1* and *jag1b* g3′UTR mutants shown as Fig. 5c (3 independent experiments; ordinary one-way ANOVA). Percentage of ECs (kdrl^+^,cmyb^-^) in S phase (edu^+^) (n = WT, 19, *csnk1a1* g3′UTR, 17; *jag1b* g3′UTR, 15 embryos) and G2/M (pH3^+^) (n = WT, 17, *csnk1a1* g3′UTR, 19; *jag1b* g3′UTR, 16 embryos) at 32hpf as shown in Fig. 4c (3 independent experiments; ordinary one-way ANOVA with Tukey’s multiple comparison). (**g)** Heatmap of erythroid (*gata1a*, *hbaa1*, *hbaa2*, *hbba1* and *hbba2*), lymphoid (*ikzf1*, *hmgb2a* and *cd81a*) and myeloid (*lcp1*, *rac2, coro1a and spi1b*) defining markers in C8 and C5 WT, *csnk1a1* and *jag1b* g3′UTR mutants. (**h-j**) UMAP showing expression of erythroid markers (*gata1a* and *alas2*) (**h**) within C8.nHSPC pLEPs and lymphoid markers (*hmgb2a* (**i**) and *ikzf1* (**j**)) within C8.nHSPC pLEPs and C5.nHSPC pLMPs. (**k,l**) UMAP and violin plot showing expression of myeloid marker *lcp1* (**k**) and *spi1b* (**l**) in C8.nHSPC pLEPs and C5.nHSPC pLMPs (Mann-Whitney test). Erythroid markers are enriched in C8 of *csnk1a1* g3′UTR, lymphoid markers are enriched in both C8 and C5 of *jag1b* g3′UTR, while myeloid markers are unchanged in both g3′UTR mutants. All quantification are represented with mean ± SEM. ns: p > 0.05, **p ≤ 0.01, **** p ≤ 0.0001. Abbreviations: primed lympho-erythroid progenitors (pLEPs) and primed lympho-myeloid progenitors (pLMPs).

**Extended Data Fig. 7:**
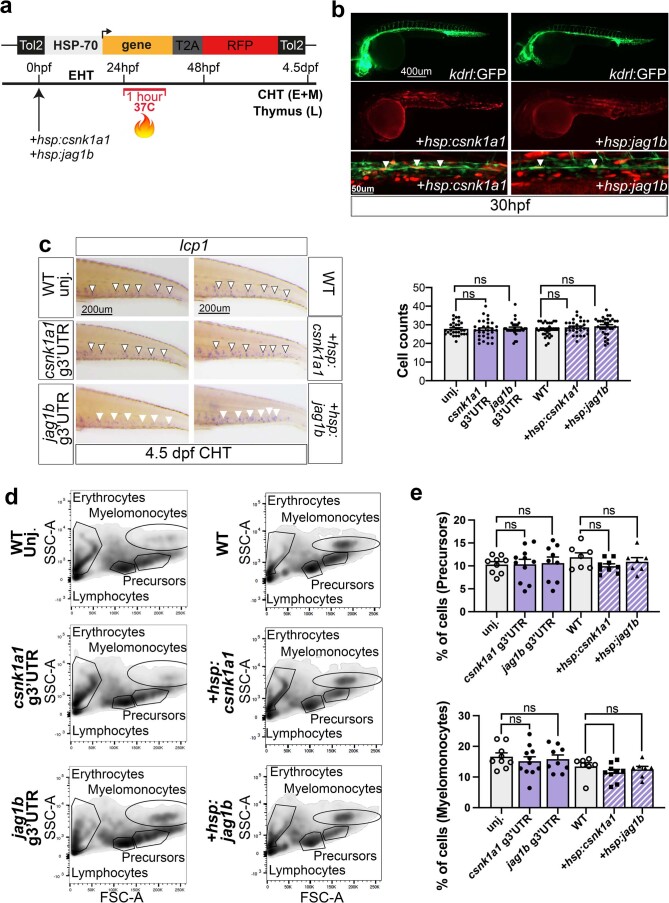
*miR-128 regulation of csnk1a1 and jag1b differentially drive erythroid and lymphoid blood lineages*. (**a**) Schematic representation of heat-shock strategy to temporally and transiently express *csnk1a1* or *jag1b* in EHT (24-30hpf). WT Tg(*kdrl:GFP*^*zn1*^) embryos were injected at one cell state with transposase and Tol2 *hsp*:*jag1b-T2A-RFP or* Tol2 *hsp*:*csnk1a-T2A-RFP*. Heat-shock (Methods) were performed at 24 hpf and blood progenitors analyzed on RFP+ embryos at 4.5 dpf. (**b**) Fluorescence stereoscope and confocal representative images of embryos treated as in **a**, at 30hpf (3 independent experiments). (**c**) Quantification of the myeloid progenitors by *lcp1* WISH (n = WT, 30; *csnk1a1* g3′UTR, 30; *jag1b* g3′UTR, 30; WT, 31; *hsp:csnk1a1*, 30; *hsp:jag1b*, 32 embryos) at 4.5dpf of WT (3 independent experiments; ordinary one-way ANOVA). (**d**) Flow cytometry population analysis defined by SSC-A and FSC-A of ~2-month-old dissected whole KM WT, *csnk1a1*, *jag1b* g3′UTR mutants and WT, *hsp:csnk1a1* and *hsp:jag1b* after heat-shock at 24-30 hpf. (**e**) Quantification of adult blood cell population in (**d)** and Fig. 4j. Immature precursors and myelomonocytes are unchanged in any condition compared to their respective controls (n = WT, 9; *csnk1a1* g3′UTR, 11; *jag1b* g3′UTR, 9; WT, 7; *hsp:csnk1a1*, 9; *hsp:jag1b*, 7 embryos; two-way ANOVA with Tukey’s multiple comparisons). All quantification are represented with mean ± SEM. ns: p > 0.05. Abbreviations: CHT: Caudal hematopoietic tissue, FSC: Forward scatter and SSC: side scatter.

To examine nHSPC heterogeneity in the AGM of each g3′UTR mutant, we performed scRNA-seq of *kdrl+* tail cells at 26 hpf (Extended Data Fig. 6a–c and Supplementary Table 4a). Notably, both *csnk1a1* g3′UTR and *jag1b* g3′UTR mutants showed an expansion of *gata2b+* cells in pre-EHT clusters, as well as of the *cmyb+* cells C8.nHSPC pLEPs and C5.nHSPC pLMPs, whereas C3.nHSPCs were expanded only in *csnk1a1* g3′UTR (Extended Data Fig. 6d,e and Supplementary Table 4b,c). Accordingly, *kdrl+cmy*+ nHSPCs were expanded in both mutant AGMs; however, *csnk1a1* g3′UTR nHSPCs were mainly replicating (EdU+), whereas those in *jag1b* g3′UTR were mainly in G2/M (pH3+) (Fig. 5c and Extended Data Fig. 6f). Next, we analysed the transcriptomes of the expanded C8.nHSPC pLEPs and C5.nHSPC pLMPs, and found that de-repression of *csnk1a1* had a large effect on differential gene expression in pLEPs, which showed upregulation of multiple erythroid markers (Fig. 5d,e and Extended Data Fig. 6g,h). In contrast, de-repression of *jag1b* led to differential gene expression in both pLEPs and pLMPs and the upregulation of multiple lymphoid markers (Fig. 5f,g and Extended Data Fig. 6g,i,j). No difference was detected for myeloid progenitor markers (Extended Data Fig. 6g,k,l). To determine the consequences on blood progenitors and blood lineages, we examined the g3′UTR mutants and also used a temperature inducible system to upregulate *cskn1a1* and *jag1b* during EHT, at 24 hpf (Extended Data Fig. 7a,b). Notably, in both cases, increased *csnk1a1* expression resulted in an expansion of erythroid progenitors in the CHT at 4.5 dpf and erythrocytes in 1-month-old WKM without altering the number of lymphoid and myeloid progenitors and relative lineages (Fig. 6a–c and Extended Data Fig. 7c–e). Conversely, in both cases, increased *jag1b* expression resulted in an expansion of lymphoid progenitors in the thymus at 4.5 dpf and lymphocytes in the 1-month-old WKM without altering the number erythroid, myeloid progenitors and their blood lineages (Fig. 6a–c and Extended Data Fig. 7c–e).

Overall, these results suggest that nHSPC heterogeneity established in the embryonic EHT by miR-128-mediated regulation of Wnt and Notch affects long-term production of erythroid and lymphoid lineages (Fig. 6d,e).

## Discussion

In this study, we discovered regulatory networks in vasculature that govern definitive nHSPC heterogeneity during embryonic EHT. This finding suggests that nHSPCs inherit distinct behaviours, such as cell cycle states and lineage priming, from AGM endothelium that influence blood composition in both embryo and adult. Our data suggest that different nHSPC primed phenotypes originate from the balance of Wnt and Notch signalling in ECs before haemogenic/HSPC specification. Mechanistically, we showed that miR-128 post-transcriptional inhibition of the canonical Wnt inhibitor *csnk1a1* limits the formation in the AGM of replicative nHSPCs, and of erythroid-biased nHSPCs, erythroid progenitors and erythrocytes in secondary and definitive haematopoietic organs. On the other hand, the miR-128 repression of the Notch ligand *jag1b* limits the generation of G2 and lymphoid-biased nHSPCs in the AGM and lymphoid progenitor cells and lymphocytes. Thus, regulation of Wnt signalling and regulation of Notch signalling in the AGM have opposing activities in nHSPCs cell cycle and differentiation outputs, influencing adult definitive organ composition.

Classically, HSCs have been considered discrete homogeneous populations, and blood formation was thought to occur through a stepwise progression of HSCs from multi- (multilineage potential), to oligo- (lineage-restricted potential), to unipotent (single-lineage potential) progenitors, to mature blood cells, following a tree-like hierarchy^54,55^. New insights from transcriptomics^12,56–59^, genetic lineage tracing^4,60^ and transplantation studies^61–63^ propose that HSC and multipotent progenitor types are intrinsically heterogeneous, with HSCs/MMPs lying along a continuum of states rather than a stepwise hierarchy^11,64^, transforming the classical view of HSPC lineage commitment^64–66^. Furthermore, embryonic HSC/MMP phenotypes in the AGM can functionally influence the blood composition of young adult animals^2,5^. Whether the diverse nHPSC populations we identified here correspond to embryonic HSCs/MMPs will require further analysis. Nevertheless, our discovery suggests that their formation is regulated by signalling in the endothelium, before haemogenic specification, where for instance Notch or Wnt activity could regulate the activation of competency mechanisms leading to the diversification of haemogenic cells^16,67^ towards specific HSPC phenotypes. This could be used to optimize the dosage of Wnt or Notch molecules often used in the protocol for engineering nHSPCs into mature erythrocytes and lymphocytes, such as T cells, for example, during ex vivo production, for CAR-T cell manufacture^68^.

Heterogeneity in HSPCs has been observed in adult bone marrow. Interestingly, the regulation of HSPC heterogeneity is strongly associated with multisystem disease susceptibility and acquired genetic mosaicism during ageing^11,69^. Whether HSPC heterogeneity can be ‘corrected’ to improve these disease outcomes is yet to be considered since, until now, it was unclear how intrinsic HSPC heterogeneity can be regulated in the embryonic or definitive haematopoietic organs. Our discovery fills this gap of knowledge. Since AGM nHSPCs are destined to generate blood over a lifetime, our discovery suggests that specific EC signalling can be manipulated to either rebalance blood and immune cells or increase the production of one blood lineage versus another at birth. Further investigation will be critical to elucidate for example how the production of nHSPCs in specific cell cycle states influence their lineage priming in the AGM. Due to the stochasticity of gene expression during scRNA-seq we were unable to fully determine the direct correlation between cell cycle state and erythroid or lymphoid blood priming. Either way, the modulation of cell cycle and/or priming of nHSPCs in vivo and in vitro might open avenues to modulate blood production as needed, without compromising vascular niche-dependent phenotypes.

Reprogramming of somatic cells (including the endothelium) to produce HSPCs with long-term self-renewal and engraftment capacity often leads to cell products with heterogeneous composition, which is not desirable for HSPC transplantation in patients with blood cancer^70–72^. Our discovery suggests that the heterogeneity observed in ex vivo HSPC production might not be an effect of this cost and labour-intensive procedure, but an intrinsic property of HSPCs produced by the signalling activated in somatic cells. Indeed, we found that the endothelium of the AGM express inhibitory mechanisms to regulate HSPC heterogeneity, like miR-128. So far, we found that eliminating the miR-128-mediated post-transcriptional inhibition of *csnk1a1* and *jag1b*, can differentially control lineage priming bias- and cell cycle state-dependent HSPC production. For example, our scRNA-seq profile of nHSPC suggests a numerical increase in pLMP/pLEP nHSPCs in both *csnk1a1* and *jag1b g3UTR* mutants but not miR-128^Δ/Δ^, and all three mutants have distinct consequences on progenitor priming. Therefore, we suggest that miR-128 regulates other genetic circuits, beside *csnk1a1* and *jag1b*, to further control HSPCs phenotypes (for example, number of biased nHSPCs versus priming). Our work suggests that the rich arsenal of miR-128 target genes can be exploited to dissect and modulate precise nHSPC phenotypes in the AGM and to promote the balanced production of HSPCs ex vivo, the holy grail of this life-saving application.

## Methods

### Zebrafish husbandry

Zebrafish were raised and maintained at 28.5 °C using standard methods, and according to protocols approved by the Yale University Institutional Animal Care and Use Committee (#2017-11473). The following zebrafish transgenic lines have been described previously: miR-128^ya315-316^ (ZDB-CRISPR-161031-5 and ZDB-CRISPR-161031-9) referred to as miR-128^Δ/Δ^, Tg(kdrl:gfp^zn1^) (ZDB-ALT-070529-1, referred to as kdrl:GFP+), Tg(runx1:eGFP^y509^) (ZDB-ALT-170717-3, referred to as runx1:GFP), Tg(kdrl:hras-mCherry^s896^) (ZDB-ALT-081212-4 referred to as kdrl:mCH+); Tg(cmyb:GFP^zf169^) (ZDB-ALT-071017-1, referred to as cmyb:GFP+), Tg(7xTCF-Xla.Sia:NLS-mCherry^ia5^) (ZDB-TGCONSTRCT-110113-2 referred to as TCF:NLS-mCh) and (Tg:TP1:eGFP^um14^) (ZDB-ALT-090625-1 referred to as TP1:eGFP). Zebrafish embryos and adults were genotyped^73^ using GeneMarker (v2.4.0), with primers listed in Supplementary Table 5.

### Morpholino experiment

Antisense morpholino were synthesized by Gene Tools. Morpholino solution was injected into single-cell embryos at a final concentration of 0.75 ng μl^−1^: control morpholino (5′-CCTCTTACCTCAGTTACAATTTATA-3′), mixture of miR-128-1 (5′-ACCGGTTCACTGTGAGAAAGCCTAC-3′) and miR-128-2 (5′-ACCGGTTCACTGTGAGACGAGT-3′).

### FACS

For all the experiments, 26 hpf embryos were anaesthetized with 1× tricaine. Whole embryos, tails or head-dissected embryos were placed in phosphate-buffered saline (PBS) 1× (pH 7.4, Invitrogen) and were dissociated into single-cell suspensions through treatment with liberase enzyme (Roche) for 1 h at 28 °C. Liberase was then inactivated with foetal bovine serum (Thermo Fisher), and cell suspension was washed with cell suspension medium (0.5% foetal bovine serum, 0.8 µM CaCl_2_, 1% penicillin, Leibovitz medium L15 (Gibco))^74^. 4′,6-Diamidino-2-phenylindole (DAPI) was added to the single-cell suspension to distinguish alive cells. We recovered 92–98% alive cells and 1–2% endothelial fluorescence-activated-sorted cells in pre-coated 1.5 ml tubes with 0.04% bovine serum albumin in PBS.

### qRT–PCR

Embryos were processed whole or dissociated into single-cell suspensions and subjected to FACS. RNA was extracted from ~300,000 to 600,000 cells using Trizol (Ambion) and 300–500 ng of total RNA was used in mRNA reverse transcription reactions (Superscript 4, Thermo Fisher) and the resulting complementary DNA was used as template for SYBR Green-based quantitative PCR (Kapa Biosystems). A total of 100 pg to 10 ng of total RNA was used in miRNA reverse transcription (MirCury LNA miRNA PCR assays, Qiagen), cDNA was used as template for the SYBR Green-based quantitative PCR (MirCury LNA SYBR Green PCR, Qiagen). U6 primers (U6 snRNA), zebrafish miR-128 (dre-miR-128-3p miRCURY LNA miRNA PCR assay) and human miR-128 (hsa-miR-128-3p miRCURY LNA miRNA PCR assay) were used as commercially provided from Qiagen. The 2^−CT^ method was used to determine relative gene expression for qRT–PCR analyses. Fold change is the mRNA levels normalized to the β-actin housekeeping gene, *actb1* and was relative to the indicated control, while mature miRNA expression was normalized to U6 snRNA levels and relative to the indicated control. All primers are listed in Supplementary Table 5.

### Immunofluorescence

Immunofluorescence (IF) was performed in all zebrafish stages as follows: after overnight 4% paraformaldehyde (Santa Cruz) fixation at 4 °C, embryos were washed with 1× PBS–0.01%Tween-20 (PBSTw) four to six times for 5 min, and then permeabilized with 0.125% trypsin (Millipore Sigma T4549) for 3 min. Embryos were washed in blocking solution (0.8% Triton-X, 10% normal goat serum, 1% bovine serum albumin and 0.01% sodium azide in PBSTw) three times for 5 min, plus an additional 2 h incubation with shaking. Antibody concentrations used were 1:300 chicken anti-GFP (Abcam, ab13970, RRID: AB_300798), rabbit anti-RFP (Antibodies Online, ABIN129578, RRID: AB_10781500) or phospho-histone H3 (ref. ^75^) (pH3-ser10) mouse monoclonal antibody (Cell Signaling, #9706, RRID:AB_331748) primary antibodies and 1:400 Alexa Fluor 488 goat anti-chicken IgG165 (Thermo Fisher Scientific, cat. no. A-11039, RRID:AB_2534096) or Alexa Fluor 546 donkey anti-rabbit IgG (Thermo Fisher Scientific, cat. no. A10040, RRID:AB_2534016) secondary antibodies. Following each overnight antibody incubation at 4 °C, six washes for a total of 4 h were performed with blocking solution lacking goat serum at room temperature and then stained as stated above by IF with the addition of DAPI staining (1:500).

### EdU incorporation assay

Click-iT EdU Alexa Fluor 647 kit (Thermo Fisher, C10340) was used to analyse S phase endothelial and positively expressing kdrl+cmyb+ cells in the 32 hpf AGM. Embryos were injected at 32 hpf with 10 mM EdU staining solution^76^ into the sinus venosus and incubated for 5 min at 28 °C, followed by 4% paraformaldehyde overnight fixation at 4 °C. Embryos were washed three times for 5 min with PBSTw and placed in cold 100% acetone at −20 °C for 7 min and rinsed with dH_2_O. Subsequently, embryos were permeabilized with 1% DMSO, 1% Triton in 1× PBS for an hour and washed three times for 5 min with PBSTw and then incubated with reaction cocktail (1× reaction buffer, CuSO_4_ solution, Alexa Fluor azide and reaction buffer additive) for 1 h at room temperature in the dark. Samples were rinsed five times for 5 min with PBSTw, and then stained as stated above by IF with the addition of DAPI staining (1:500).

### Whole mount in situ hybridization

Whole mount in situ hybridization (WISH) with riboprobes against *gata2b*, *runx1*, *cmyb*, *gata1a*, *ikaros*, *lcp1*, *rag1*, *notch3*, *flt4*, *etv2* and *scl* was performed as previously described^73^. Briefly, embryos were fixed in paraformaldehyde 4% overnight and washed in methanol. Embryos were kept at −20 °C. Embryos were then rehydrated with PBSTw and permeabilized with 10 µg ml^−1^ proteinase K (Roche) (10 min for 24 hpf, 13 min for 32 hpf, 1 h for 4.5 dpf), followed by a post-fixation in paraformaldehyde 4% for 20 min. Then embryos were incubated with the specific riboprobes overnight (or over 2 days for embryos at 4.5 dpf) at 65 °C. Finally, after extensive wash, embryos were incubated with anti-DIG antibody 1:10,000 (Roche, cat. no. 11207733910). Imaged embryos were quantified as follows: *gata2b*, *cmyb*, *runx1* stained cells were counted in the region of the dorsal aorta above the yolk extension; *lcp1* and *gata1a* stained cells were counted in the CHT at 4.5 dpf. *Ikaros*, *rag1* and *cmyb* staining at 4.5 dpf and 3 or 6 dpf were quantify as area of staining using ImageJ. Bright-field images of WISH staining were acquired with a Leica Microsystems M165FC stereomicroscope equipped with Leica DFC295 camera.

### Kaede photoconversion

*Tg(fli1a1:gal4ff*^*ubs4*^*)* were outcrossed with *Tg(UAS:Kaede*^*rk8*^*)* and injected with either control or miR-128 morpholino at a concentration of 0. 75 ng μl^−1^ at the one cell stage. Embryos were screened at 24 hpf for Kaede positive, and the ventral wall of the dorsal aorta was photoconverted with a Zeiss LSM 980 scanning confocal using a 406 nm laser at an intensity of 16% for 10 s at 30 hpf followed by incubation at 28 °C. At 4.5 dpf the thymus and CHT of photoconverted embryos were imaged with a Zeiss LSM 980 confocal. Red-Kaede-positive cells representing erythroid and myeloid progenitors were counted in the CHT and red-Kaede-positive cells representing lymphoid progenitors were counted in the thymus. Red-Kaede volume in the thymus was measured using IMARIS software (V.9.9.1, Bitplane) by utilizing the surface module to create a 3D reconstruction. Red-Kaede cells in the CHT were counted using ImageJ.

### Time-lapse video

Zebrafish embryos were treated with 0.003% 1-phenyl-2-thiourea (Sigma P7629) starting at 70%/80% gastrulation stage to prevent pigmentation. Embryos imaged live by confocal microscopy were anaesthetized in 0.1% tricaine and mounted in 1% low-melting-point agarose. Fluorescent images and time-lapse movies were captured using Zeiss LSM 980 confocal microscope (Software Zeiss ZEN 3.4 (blue edition)) using 20× water immersion objective. Confocal time-lapse movies were performed at room temperature starting at 27 hpf with *z*-stacks acquired at an interval of 12 min for a total of 15 h. Time of delamination was quantified for *cmyb:GFP*+ *kdrl:mCherry*+ cells transitioning from flat morphology until they exit the ventral dorsal aorta wall into the subaortic space. Cells were tracked using dragon-tail analysis in IMARIS software (V.9.9.1, Bitplane).

### Flow cytometry analysis

WKM of ~1- or 2-month-old zebrafish were mechanically dissociated as in refs. ^77,78^. DAPI was used to differentiate alive cells. Cells were differentiated by light scatter characteristics as follow (BD FACSDiva v9.0 Software): forward scatter (FSC)^low^ correspond to mature erythroid cells; FSC^high^ and side scatter (SSC)^high^ correspond to myelomonocytes (including neutrophils, monocytes, macrophages and eosinophils)^34^; FSC^intermediate(int)^ and SSC^low^ contain lymphocyte cells (B lymphocytes, lymphoid precursors and rare HSCs)^34^. FSC^int^ and SSC^int^ contain immature precursors (myeloid, lymphoid and erythroid precursors)^34^. Tg(kdrl:gfp^zn1^) 24 hpf were dissociated to single-cell suspension using the protocol for FACS as above. DAPI was used to differentiate alive cells. Cells were analysed using a LSR Fortessa (BD Biosciences). All quantification were carried out using FlowJo software (v10.5) (Extended Data Fig. 7).

### Plasmid expression constructs

The miR-128 endothelial expression plasmid was constructed as previously described^73^. To generate the pME-miR-128 middle entry cassette for Gateway-compatible cloning, a 365 bp genomic sequence containing the miR-128-1 stem–loop precursor was PCR amplified with flanking KpnI and StuI sites, and the resulting fragment was restriction digested and cloned into pME-miR (p512 addgene) using T4 ligation (NEB M0202S) according to the manufacturer’s protocol. Promoter entry cassette p5E-fli1a (Addgene #31160), pME-miR-128 and pTol2 entry vector was combined in LR multisite Gateway cloning reaction^73^ to produce pTol2 fli1a:mCherry-miR-128 constructs. Embryos were injected in the one-cell state with 25 pg of the expression construct and Tol2 transposase mRNA, and later selected for mCherry expression.

Both *csnk1a1* and *jag1b* middle entry vectors were synthesized commercially by GeneArt (Thermo Fisher) and inserted into pDONR221 utilizing the coding sequence of *csnk1a1* (ENSDART00000121429.4) and *jag1b* (ENSDART00000019323.7) lacking their STOP codon. The subsequent plasmids were recombined with p5E-*Hsp70* promoter, p3E-T2A-RFP and pDESTtol2pA utilizing LR multisite Gateway cloning. All Tol2-based plasmid were injected with Tol2 transposase mRNA into single-cell embryos at 25 pg per embryo. Injected embryos with *hsp:csnk1a1* and *hsp:jag1b* were dechorionated and treated with heat shock in a water bath for 1 h at 37 °C followed by a 15 min room temperature incubation and stored at 28 °C for further development until 4.5 dpf or 1 month old. Embryos were screened for RFP expression 2 h post heat shock treatment.

### gRNA generation and Cas9 injection

CRISPRScan (https://www.crisprscan.org) was used to design guide RNAs (gRNAs) to mutate the miR-128 responsive element region in *jag1b* and *csnk1a1* 3′UTRs. gRNA preparation was performed as previously^79^. WT embryos were injected with 100 pg of gRNAs and 200 pg of *Cas9* mRNA at the one-cell stage. PCR genome amplification and T7E1 assay was used to validate indels as previously^79^. Sequences and primers are listed in Supplementary Table 5 and Extended Data Fig. 7.

### Bulk and scRNA-seq sample preparation

To identify the vascular transcripts regulated by miR-128 for bulk RNA-seq we prepare three replicates of *Tg(kdrl:GFP*^*zn1*^*)* WT and miR-128^Δ/Δ^ tail *kdrl:GFP*^+^ ECs isolated via FACS from 26 hpf. Total RNA was then isolated with the Lexogen SPLIT RNA Extraction Kit, and ∼10 ng was used to prepare Lexogen QuantSeq 3′ mRNA-Seq libraries for Illumina deep sequencing according to the manufacturer’s protocol. Libraries were amplified with ∼17 PCR cycles using the Lexogen PCR Add-on Kit according to the Lexogen manufacturer’s protocol.

CD34+CD43− hPSC-derived cells were sorted on day 8 of differentiation and were RNA extracted with TRIzol. Libraries (tail: 3 for WT and 4 for miR-128^Δ/Δ^, head: 2 for WT and 4 for miR-128^Δ/Δ^) were then prepared the same way as zebrafish cells. A total of 5 ng of RNA was used in each sample, and 19–21 cycles were used for library amplification. Libraries were then deep sequenced according to the Illumina manufacturer’s protocol on an Illumina Hiseq 2500, at the Yale Center for Genome Analysis. For scRNA-seq WT, miR-128^Δ/Δ^, *csnk1a1* and *jag1b* g3′UTR mutants *Tg(kdrl:GFP*^*zn1*^*)* tail tissue containing the AGM were dissected at 26 hpf. Kdrl:GFP+ cells, which had >85% cell viability, were loaded onto the 10x Genomics Chromium instrument for a targeted recovery of 10,000 cells per sample. The 10x Genomics Chromium Next GEM Single Cell 3′ Library Construction Kit V3.1 (CG000204) was used to generate libraries according to manufacturer instructions. Barcoded libraries were sequenced on an Illumina HiSeq 4000 instrument.

### Bulk RNA sequencing bioinformatic analysis

Bulk RNA sequencing was analysed with principal workflow demonstrated by Lexogen. Freely available tools were part of the Galaxy platform^80^. Specifically, quality of data was checked with FastQC^81^. BBDuk was used to remove the adaptor contamination, polyA readthrough and low-quality tails^82^. Zebrafish genome index was generated with STAR^83^ according to GRCz10 (Ensembl release 91)^84^, and decontaminated reads were mapped to the zebrafish genome^85^. Output BAM files were indexed with SAMtools^86^. Reads were counted with HTSeq^87,88^. Genes below five read counts in all replicates in either condition were filtered out with a customized Python script. Differentially expressed genes between WT and miR-128−/− mutant conditions were identified with DESeq2^89^. Significantly differentially expressed genes in miR-128−/− tail and head ECs were examined for miR-128 binding sites with TargetScanFish Release 6.2^90^. Kyoto Encyclopedia of Genes and Genomes (KEGG) pathway terms were assigned to differentially expressed genes with DAVID^91,92^. Differentially expressed miR-128 target genes, KEGG pathways can be found in Supplementary Table 2. hPSC samples were processed using a similar pipeline as described for zebrafish samples above. No Poly-A sequence removal was necessary. Ensemble genome reference build GRCh38 was used. WNT- and NOTCH-regulated genes are presented in Supplementary Table 4.

### scRNA-seq bioinformatic analysis

#### scRNA-seq, quality control

RNA sequencing quality assurance was performed using FastQC (version 0.11.9), by looking for the presence of adapters and sequence quality through Phred Score. Genome alignment was performed using the 10x Genomics Cell Ranger pipeline (version 5.0.0). A transcriptome reference using a customized zebrafish genome annotation^93^ was built that corrected 3′UTR annotation problems and improved alignment performance. The resulting filtered feature-barcode matrices were used for downstream analysis.

The filtered count matrices were loaded on RStudio (version 4.1.1), and the Seurat (version 4.0.6) class object was used to store the data. Cells with fewer than 200 features and features detected in fewer than three cells were removed. Cell quality control was performed looking at the overall distribution of counts, detected genes and expression of mitochondrial genes. Cell doublets, cells having more than 35,000 unique molecular identifiers, were removed. After those quality controls, 22,230 cells (WT and miR-128^Δ/Δ^ cells) were kept.

Each sample was integrated, and batch effect correction was performed using an algorithm based on mutual-nearest neighbours, which finds shared cell populations across different datasets and creates anchors to remove non-biological signals^94^. For this, data were normalized with NormalizeData and 2,000 highly variable genes (HVGs) were identified. These HVGs were used to define the integration anchors. The first 20 dimensions were used to perform the integration. For scRNA-seq data analysis, uniform manifold approximation and projection (UMAP) was applied to the integrated data to obtain a representation of the manifold. The neighbourhood graph was calculated using FindNeighbors, and clusters were extracted using FindClusters at the resolution of 0.5, both functions defined on Seurat. Cell type identities were assigned to clusters through visualization of canonical cell type markers and differential gene expression. Gene markers defining each cell cluster regardless of cell genotype were identified using the FindConservedMarkers function implemented on Seurat. EHT cell clusters, 6,096 cells were then subset and reclustered. The cell subset was re-analysed, rescaled and normalized, new HVGs were identified and new UMAP with a resolution of 0.3 was produced.

#### Pseudotime analysis

Monocle 3 find_gene_modules function was employed to explore cell identities by finding modules of genes co-expressed across cells^95^. Then, g:Profiler was used to functionally characterize the modules through an enrichment analysis. Cell trajectory was also reconstructed by calculating the pseudotime value having as root or starting point the pre-hem C1 population.

#### RNA velocity analysis

scVelo (version 0.2.3) was used for modelling RNA velocity, employing an algorithm that generalizes splicing kinetics for different cell populations instead of assuming different populations share the same splicing ratio^96^. We used Velocyto (version 0.17.17) to obtain the unspliced count matrix for each sample^97^. The count matrices were integrated into the data using the Anndata structure on PyCharm (version 2021.1).

CellRank (version 1.4.0) was employed as an additional analysis for exploring cell trajectory. This algorithm addresses the noise in RNA velocity data by integrating other sources of input data such as cell transcriptomic similarities^98^. A transition probability towards a detected terminal state is calculated for each cell. The transition probabilities were calculated for the reclustered dataset containing WT and 128^Δ/Δ^ cells.

#### Cell cycle analysis

Cell cycle phases were characterized by performing a cell cycle scoring analysis using Seurat’s function CellCycleScoring with cell cycle phase markers^99^. Next, cell phases were transformed into numeric values and one-way analysis of variance (ANOVA) was performed on the cluster-genotype groups to identify if there were changes in cell phase distribution across them. To identify group changes, multiple Student’s *t*-test were executed and the Bonferroni post-hoc correction for multiple testing was employed.

#### Wnt and Notch signalling signature analysis

AUCell (version 1.14.0) was employed to analyse WT and miR-128^Δ/Δ^ cells. The area under the curve was used to quantify and test the signature enrichment of Wnt and Notch gene sets in each cell. The gene sets used were described in the KEGG for both Notch (*dre04330*) and Wnt (*dre04310*).

#### Analysis of csnk1a1 and jag1b g3′UTR RNA samples

The *csnk1a1 and jag1b g3*′*UTR* single-cell samples were processed similarly to the previous ones and resulted in 20,600 kdrl+ cells. Clustering analysis was performed using a resolution of 0.5 and EHT clusters were reclustered, accounting for a total of 7,949 cells. Cells were projected on the reclustered UMAP and their identities were predicted using our previous cluster-cell type annotation using Seurat MapQuery function. Then, cells were filtered on the basis of their cell type prediction score, removing cells with a max.prediction.score lower than 0.55, followed by a new round of projection. A specific filter (prediction.score >0.70) was applied specifically in the cells from clusters 7 and 8 removing cells sparsely distributed throughout the UMAP visualization. In total, 5,782 cells were kept.

#### MELD analysis

MELD was employed (standard parameters) for quantifying the effect of experimental perturbations at the single-cell resolution for a mutant dataset compared with a reference one^100^.

All the statistics analysis comparing number of cells and expression level (violin plots) are done through comparing a total number of cells in each condition allowing statistical analysis. Statistical tests used in each experiments are specified in the legends.

### hPSC differentiation procedure

#### Maintenance and differentiation

The hESC H1 line (WiCell, #WA01) was maintained on irradiated mouse embryonic fibroblasts in hESC medium^101,102^. For differentiation, hPSCs were cultured on Matrigel-coated plasticware (Corning Life Sciences) for 24 h, followed by embryoid body (EB) generation^103–105^. Briefly, hPSCs were dissociated with brief trypsin-EDTA (0.05%) treatment, followed by scraping. For the first 3 days of differentiation, EBs were resuspended in SFD medium^106^ supplemented with l-glutamine (2 mM), ascorbic acid (1 mM), monothioglycerol (MTG, 4×10-4M; Sigma), and transferrin (150 μg ml^−1^). On day 0, EBs were treated with BMP4 (10 ng ml^−1^). Twenty-four hours later, basic fibroblast growth factor (bFGF) (5 ng ml^−1^) was added. On the second day of differentiation, ACTIVIN A (1 ng ml^−1^), SB-431542 (6 μM), CHIR99021 (3 μM) and/or IWP2 (3 μM) were added, as indicated. On the third day, differentiation cultures were changed to StemPro-34 medium supplemented with l-glutamine, ascorbic acid, MTG and transferrin, as above, with additional bFGF (5 ng ml^−1^) and VEGF (15 ng ml^−1^). On day 6, IL-6 (10 ng ml^−1^), IGF-1 (25 ng ml^−1^), IL-11 (5 ng ml^−1^), SCF (50 ng ml^−1^) and EPO (2 U ml^−1^ final). All differentiation cultures were maintained at 37 °C. All embryoid bodies and mesodermal aggregates were cultured in a 5% CO_2_/5% O_2_/90% N_2_ environment. All recombinant factors are human and were obtained from Biotechne. Analysis of haematopoietic colony potential was performed via Methocult (H4034; Stem Cell Technologies)^103,107^.

#### miR-128 manipulation within cultures

Cells were left untreated (vehicle control) or treated with antagomiR specific to miR-128 or a scramble antagomir (100 μM in molecular grade water) to a final concentration of 200 nM.

#### Flow cytometry and cell sorting

Cultures were dissociated to single cells, as previously described^102^. Cells were washed, labelled, sorted and collected in StemPro-34 medium. The antibodies used are: mouse anti KDR-PE (0.25 µg per 10^6^ cells, Biotechne, cat. no. MAB3572 clone 89106), mouse anti CD34-PE-Cy7 (BD, cat. no. 348791 clone 8G12), mouse anti CD43-FITC (BD, cat. no. 555475 clone 1G10), mouse anti CD73-PE (BD, cat. no. 550257 clone AD2), mouse anti CXCR4-APC (BD, cat. no. 555976 clone 12G5) and mouse anti CD235a-APC (BD, cat. no. 551336 clone HIR-2) (refs. ^102,103,107^). All antibodies were obtained from BD Biosciences except for KDR (Biotechne). Cells were sorted with a FACSMelody (BD) cell sorter (Source data). For isolation of mesodermal populations, day 3 of differentiation HOXA+KDR+CD235a− or HOXA−KDR+CD235a+ were FACS-isolated and re-aggregated at 250,000 cells ml^−1^ in day 3 medium, as above. Cultures were plated in 250 μl volumes in a 24-well low-adherence culture plate, and grown overnight in a 37 °C incubator, with a 5% CO_2_/5% O_2_/90% N_2_ environment. On day 4, an additional 1 ml of antagomiR- or scramble-supplemented day 3 medium was added to re-aggregates. On day 6 of differentiation, cultures were fed as normally and subsequent CD34+CD43− cells were sorted on day 8 of differentiation.

#### Haemato-endothelial growth conditions of hPSC-derived haemogenic endothelium

Either CD34+CD43− cells (stage 1 antagomir manipulation) or CD34+CD43−CD73−CD184− cells (stage 2 antagomir manipulation) were isolated by FACS and allowed to undergo the EHT^105,107^. Briefly, cells were aggregated overnight at a density of 2 × 105 cells ml^−1^ in StemPro-34 medium supplemented with l-glutamine (2 mM), ascorbic acid (1 mM), MTG (4 × 10^−4^ M; Sigma-Aldrich), holo-transferrin (150 μg ml^−1^), TPO (30 ng ml^−1^), IL-3 (30 ng ml^−1^), SCF (100 ng ml^−1^), IL-6 (10 ng ml^−1^), IL-11 (5 ng ml^−1^), IGF-1 (25 ng ml^−1^), EPO (2 U ml^−1^), VEGF (5 ng ml^−1^), bFGF (5 ng ml^−1^), BMP4 (10 ng ml^−1^), FLT3L (10 ng ml^−1^) and SHH (20 ng ml^−1^). Aggregates were spotted onto Matrigel-coated plasticware and were cultured for additional 9 days. Cultures were maintained in a 37 °C incubator, in a 5% CO_2_/5% O_2_/90% N_2_ environment. All resultant cells within the haemato-endothelial cultures were subsequently collected by trypsinization, and assessed for haematopoietic potential by Methocult in a 37 °C incubator, in a 5% CO_2_/air environment.

### Statistics and reproducibility

The numerical visualizations were generated using ggplot2 (version 3.3.5) and GraphPad Prism (version 9). Sample size and statistical test are specified in each legend. Sample size in each experiment was validated via power analysis (Source data), and extreme numerical data were excluded when data were considered outliers resulting from clear technical issues. Allocation of samples to each experimental groups was randomized. Mann–Whitney test was used when comparing two groups to test mean differences (WISH, live imaging, IF and scRNA seq). Ordinary one-way ANOVA with Tukey’s multiple comparison was used when comparing more than two groups to test mean differences (WISH, IF, qRT–PCR and hPSC quantification). Two-way ANOVA with Tukey’s multiple comparisons was used to test mean differences among two or more groups under multiple conditions (WKM analysis). One-sample *t*-test and Wilcoxon test was used to compare two-paired samples (qRT–PCR). scRNA-seq experiments were performed in one individual experiment but composed of multiple biological samples (*n* = 200 (WT), 200 (128^Δ/Δ^), 180 (*csnk1a1* g3′UTR) and 170 (*jag1b* g3′UTR) embryos). All images of embryo were blinded before quantification. For hPSCs, investigator was not blinded as only one person performed the experiments. All experiments were performed with at least three independent experiments. Data distribution was assumed to be normal, but this was not formally tested.

### Reporting summary

Further information on research design is available in the Nature Portfolio Reporting Summary linked to this article.

## Online content

Any methods, additional references, Nature Portfolio reporting summaries, source data, extended data, supplementary information, acknowledgements, peer review information; details of author contributions and competing interests; and statements of data and code availability are available at 10.1038/s41556-023-01187-9.

## Supplementary information

Supplementary Information Flow cytometry original images for all experiments.

Reporting Summary

Supplementary Tables Supplementary Table 1. **a**, Average expression of markers in all cells within each EHT clusters. **b**, Gene Ontology terms analysis, obtained by DAVID bioinformatics resource, of cluster defining genes (*P* values obtained by DAVID Fisher’s exact *P* values). **c**, Total number of cells per clusters in WT and miR-128^Δ/Δ^. **d**, Raw gene expression in WT and miR-128^Δ/Δ^ in each cell. **e**, Numbers of cells defined by their cell cycle status (G1, G2/M and S phases) in each cluster in WT cells. Supplementary Table 2. **a**, Gene Ontology terms, obtained by DAVID bioinformatics resource, from differential gene expression between WT and miR-128^Δ/Δ^ tail endothelial (*kdrl*^+^) cells at 26 hpf. **b**, Gene Ontology terms, obtained by DAVID bioinformatics resource, from differential gene expression between WT and miR-128^Δ/Δ^ head endothelial (*kdrl*^+^) cells at 26 hpf. **c**, Raw gene expression of miR-128 predicted target genes part of Wnt and Notch signaling, in WT and miR-128^Δ/Δ^ ECs from tail or head bulk RNA sequencing. Supplementary Table 3. Raw gene expression of WNT- and NOTCH-regulated genes from bulk RNA-seq between scramble and antagomir-128 treated hPCS. Expression of WNT- and NOTCH-regulated genes in control, scramble or antagomiR-128 hemogenic CD34+CD43− hPSCs treated at stage 1. Supplementary Table 4. **a**, Average gene expression in *csnk1a1* and *jag1b* g3′UTR mutant kdrl+ cells per cluster. **b**, Total number of cells in each cluster per genotype (WT, miR-128^Δ/Δ^, *csnk1a1* g3′UTR and *jag1b* g3′UTR). **c**, Number of cells expressing *gata2b* in WT, miR-128^Δ/Δ^, *csnk1a1* g3′UTR and *jag1b* g3′UTR genotypes. Supplementary Table 5. List of primers used in this study.

## Data Availability

Sequencing data that support the findings of this study have been deposited in the Gene Expression Omnibus (GEO) under accession code GSE210942. Source data are provided with this paper. They can be found on figshare (10.6084/m9.figshare.22587145). All other data supporting the findings of this study are available from the corresponding author on reasonable request.

## Source data

Source Data Fig. 1 Uncropped gel images used in Extended Data Fig. 5h.
Source Data Fig. 1 Statistical source data.
