# Supplementary material for: Haematopoietic stem and progenitor cell heterogeneity is inherited from the embryonic endothelium
Source: Nat Cell Biol. 2023 Jul 17;25(8):1135–45. doi: 10.1038/s41556-023-01187-9 (PMC10415179; doi:10.1038/s41556-023-01187-9)

nature cell biology

Article https://doi.org/10.1038/s41556-023-01187-9

Haematopoietic stem and progenitor cell heterogeneity is inherited from the embryonic endothelium

In the format provided by the authors and unedited

nature cell biology

Article

https://doi.org/10.1038/s41556-023-01187-9

Haematopoietic stem and progenitor cell heterogeneity is inherited from the embryonic endothelium

In the format provided by the authors and unedited

Flow cytometry images used in Fig. 1i and Extended Data Fig. 1p

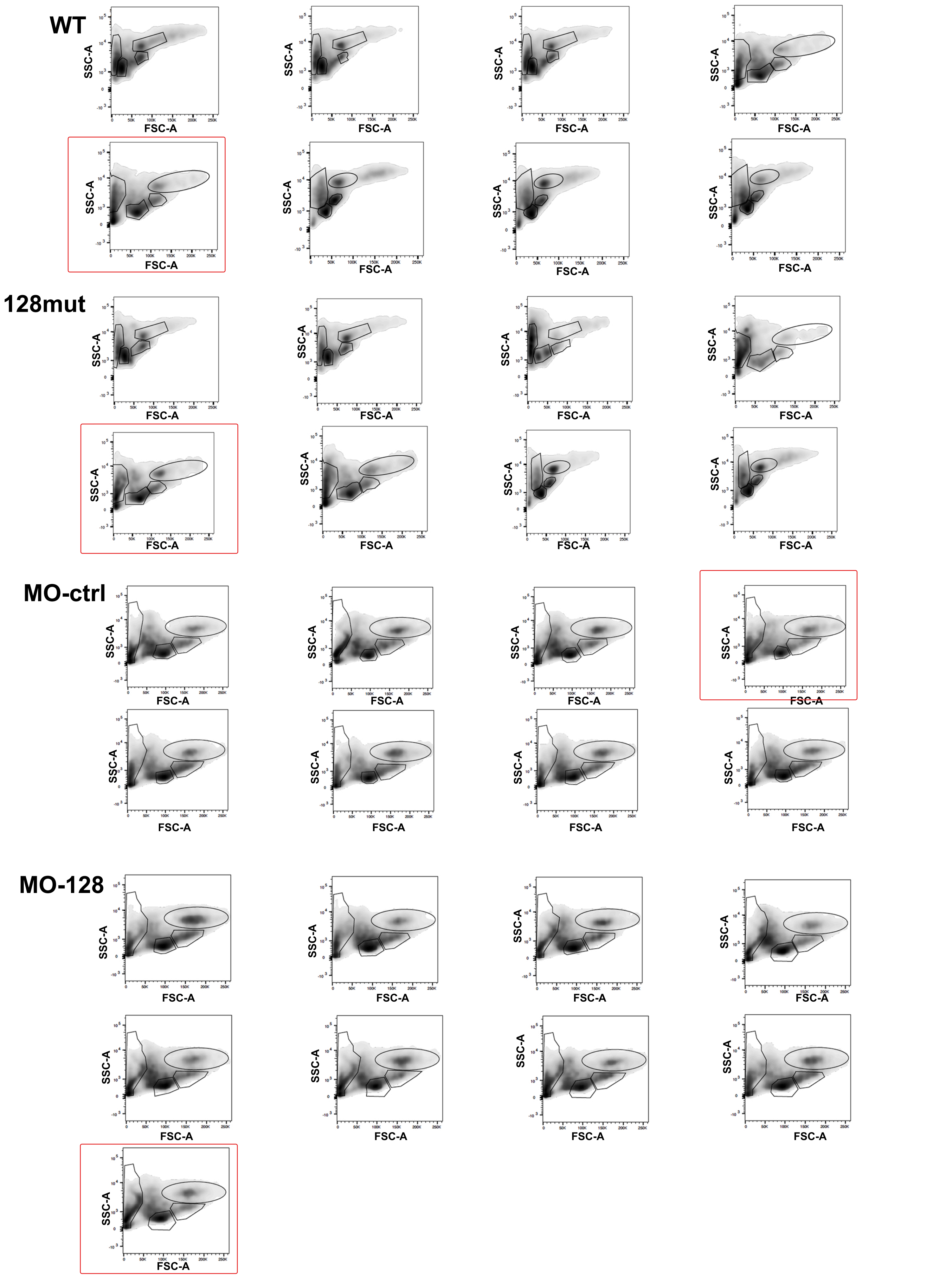

Flow cytometry images used in Fig. 6c and Extended Data Fig. 7d,e

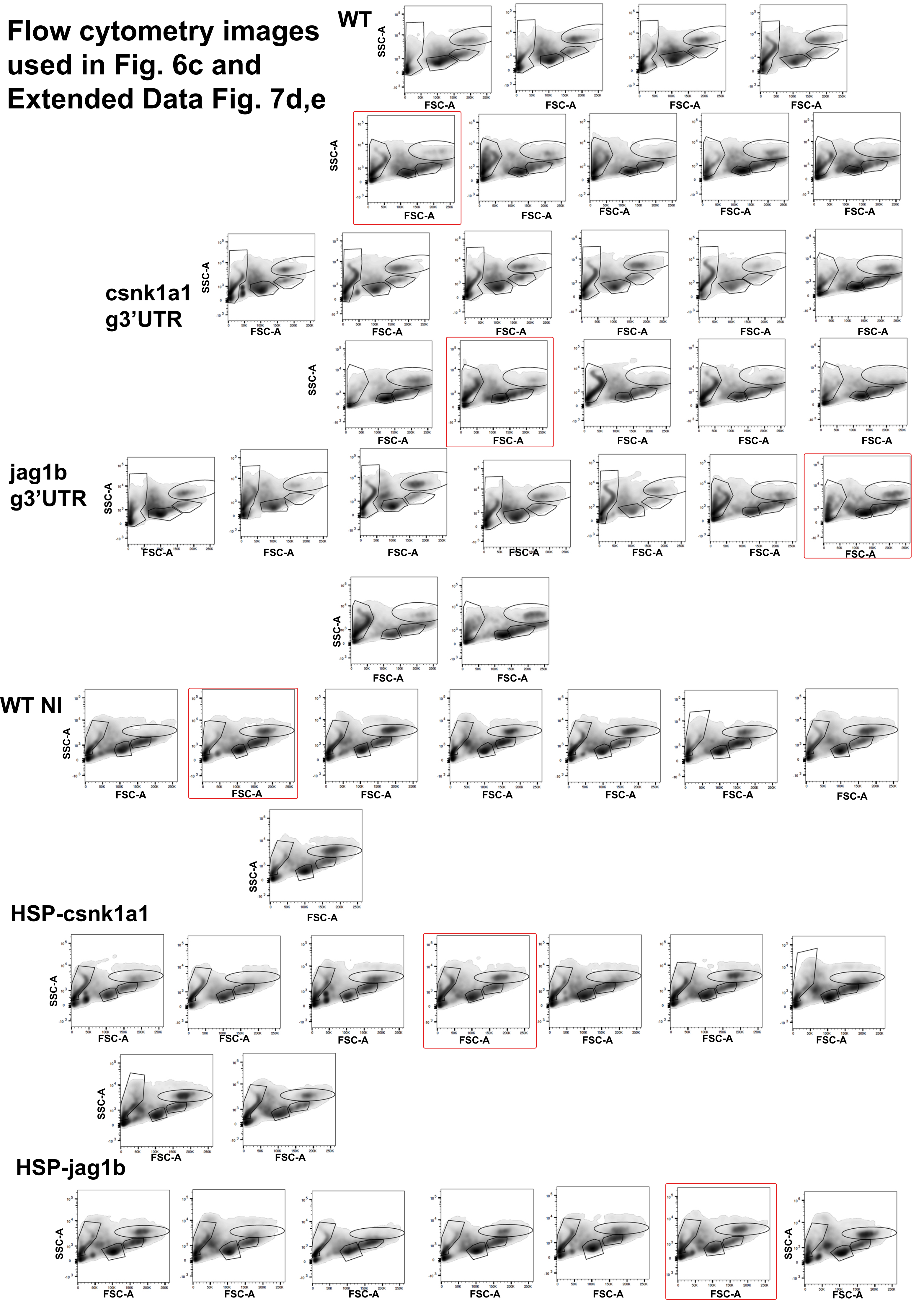

Flow cytometry images used in Extended Data Fig. 4

Stage 1

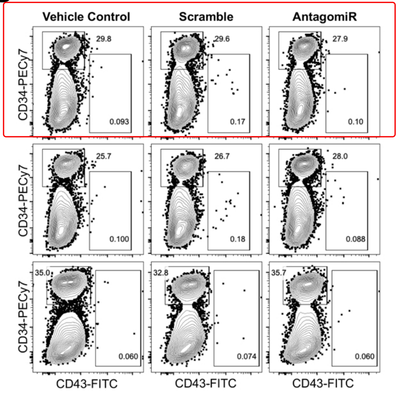

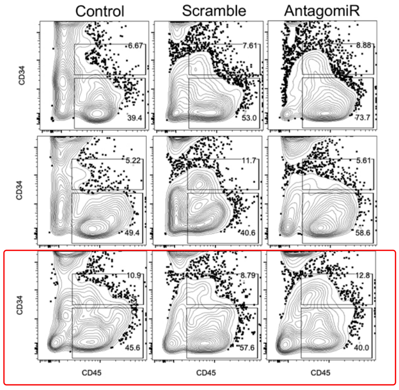

Stage 2

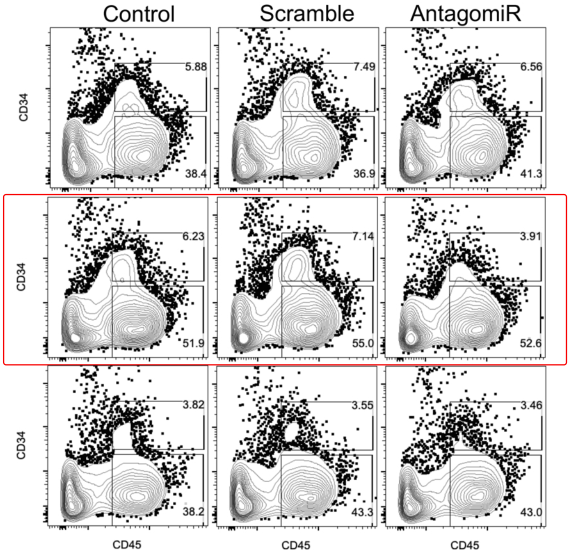

Primitive

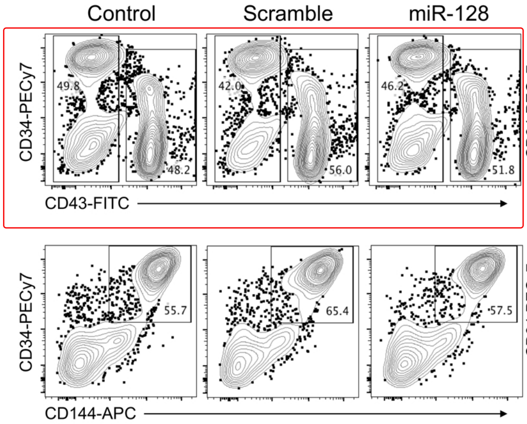

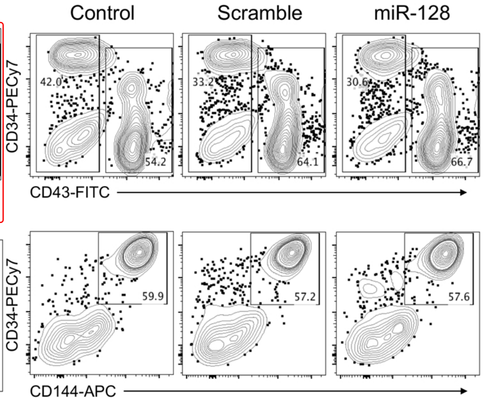

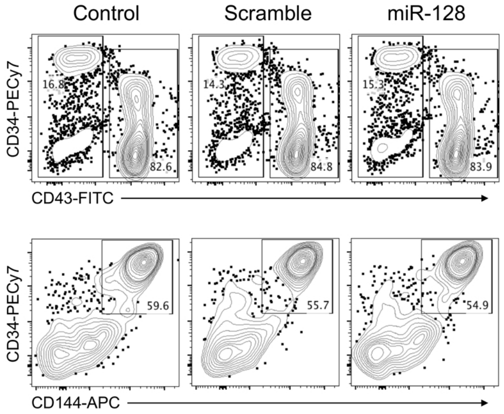

Supplement: Supplementary file 1 — Flow cytometry original images for all experiments. [file 41556_2023_1187_MOESM1_ESM.pdf]
