# Supplementary material for: Haematopoietic stem and progenitor cell heterogeneity is inherited from the embryonic endothelium
Source: Nat Cell Biol. 2023 Jul 17;25(8):1135–45. doi: 10.1038/s41556-023-01187-9 (PMC10415179; doi:10.1038/s41556-023-01187-9)
Supplement: Supplementary file 2 — Reporting Summary [file 41556_2023_1187_MOESM2_ESM.pdf]

nature portfolio

Corresponding author(s): Stefania Nicoli

Last updated by author(s): 04/11/2023

Reporting Summary

Nature Portfolio wishes to improve the reproducibility of the work that we publish. This form provides structure for consistency and transparency in reporting. For further information on Nature Portfolio policies, see our Editorial Policies and the Editorial Policy Checklist.

Statistics

For all statistical analyses, confirm that the following items are present in the figure legend, table legend, main text, or Methods section.

n/a	Confirmed	
☐	☒	The exact sample size (n) for each experimental group/condition, given as a discrete number and unit of measurement
☐	☒	A statement on whether measurements were taken from distinct samples or whether the same sample was measured repeatedly
☐	☒	The statistical test(s) used AND whether they are one- or two-sided<br>*Only common tests should be described solely by name; describe more complex techniques in the Methods section.*
☒	☐	A description of all covariates tested
☒	☐	A description of any assumptions or corrections, such as tests of normality and adjustment for multiple comparisons
☐	☒	A full description of the statistical parameters including central tendency (e.g. means) or other basic estimates (e.g. regression coefficient) AND variation (e.g. standard deviation) or associated estimates of uncertainty (e.g. confidence intervals)
☐	☒	For null hypothesis testing, the test statistic (e.g. F, t, r) with confidence intervals, effect sizes, degrees of freedom and P value noted<br>*Give P values as exact values whenever suitable.*
☒	☐	For Bayesian analysis, information on the choice of priors and Markov chain Monte Carlo settings
☒	☐	For hierarchical and complex designs, identification of the appropriate level for tests and full reporting of outcomes
☒	☐	Estimates of effect sizes (e.g. Cohen's d, Pearson's r), indicating how they were calculated

Our web collection on statistics for biologists contains articles on many of the points above.

Software and code

Policy information about availability of computer code

Data collection	GeneMarker (v2.4.0)<br>Leica LAS X Software<br>Zeiss ZEN 3.4 (blue edition) Software
Data analysis	ImageJ 1.52p<br>Graphpad Prism v 9<br>ggplot 2 v3.3.5<br>Imaris V.9.9.1<br>FlowJo software v10.5<br>BD FACSDiva v9.0 Software

For manuscripts utilizing custom algorithms or software that are central to the research but not yet described in published literature, software must be made available to editors and reviewers. We strongly encourage code deposition in a community repository (e.g. GitHub). See the Nature Portfolio guidelines for submitting code & software for further information.

Data

Policy information about availability of data

All manuscripts must include a data availability statement. This statement should provide the following information, where applicable:

- Accession codes, unique identifiers, or web links for publicly available datasets
- A description of any restrictions on data availability
- For clinical datasets or third party data, please ensure that the statement adheres to our policy

All genomic data are available in GEO with accession number GSE2109422 (scRNA-seq and bulk-RNA-seq). All numeral data for all experiments and replicas are represented in Source data and figshare (https://doi.org/10.6084/m9.figshare.22587145). All numerical data are available in Source Data files.

Human research participants

Policy information about studies involving human research participants and Sex and Gender in Research.

Reporting on sex and gender	not applicable
Population characteristics	not applicable
Recruitment	not applicable
Ethics oversight	not applicable

Note that full information on the approval of the study protocol must also be provided in the manuscript.

Field-specific reporting

Please select the one below that is the best fit for your research. If you are not sure, read the appropriate sections before making your selection.

☒ Life sciences ☐ Behavioural & social sciences ☐ Ecological, evolutionary & environmental sciences

For a reference copy of the document with all sections, see nature.com/documents/nr-reporting-summary-flat.pdf

Life sciences study design

All studies must disclose on these points even when the disclosure is negative.

Sample size	We used Power analysis for different categories of experiment to estimate the smallest, optimal and maximal number of animal needed per condition.
Data exclusions	Extreme numerical data were excluded when data were considered outliers resulting from clear technical issues.<br>When numerical data for an experiment was beyond the maximum number of animal recommended (obtained by power analysis), we blindly and randomly excluded some data.
Replication	Once protocols were established, all attempts of replication were successfully reproduced at least three times.
Randomization	Allocation of samples to each experimental groups was randomized.
Blinding	For all zebrafish experiments investigators were blinded to all group allocation prior data analysis. For hPSCs, investigator was not blinded as only one person performed the experiments.

Reporting for specific materials, systems and methods

We require information from authors about some types of materials, experimental systems and methods used in many studies. Here, indicate whether each material, system or method listed is relevant to your study. If you are not sure if a list item applies to your research, read the appropriate section before selecting a response.

Materials & experimental systems

n/a	Involved in the study
☐	☒ Antibodies
☐	☒ Eukaryotic cell lines
☒	☐ Palaeontology and archaeology
☐	☒ Animals and other organisms
☒	☐ Clinical data
☒	☐ Dual use research of concern

Methods

n/a	Involved in the study
☒	☐ ChIP-seq
☐	☒ Flow cytometry
☒	☐ MRI-based neuroimaging

Antibodies

Antibodies used	Primary and secondary antibodies used in immuno-fluorescence:<br>chicken anti-GFP (Abcam, ab13970, RRID: AB_300798)<br>rabbit anti-RFP (Antibodies Online, ABIN129578, RRID: AB_10781500)<br>phospho-histone H33 (pH3-ser10) mouse monoclonal antibody (Cell signalling, #9706, RRID:AB_331748)<br>Alexa Fluor 488 goat anti–chicken IgG165 (Thermo Fisher Scientific Cat# A-11039, RRID:AB_2534096)<br>Alexa Fluor 546 donkey anti-rabbit IgG (Thermo Fisher Scientific Cat# A10040, RRID:AB_2534016)<br>Antibody used in whole mount insitu hybridization<br>anti-DIG antibody Roche, Cat# 11207733910<br><br>Antibodies used in Human cell experiments:<br>Mouse anti KDR-PE (Biotechne Cat#MAB3572 clone 89106)<br>mouse anti CD34-PE-Cy7 (BD Cat#348791 clone 8G12)<br>mouse anti CD43-FITC (BD Cat#555475 clone 1G10)<br>mouse anti CD73-PE (BD Cat#550257 clone AD2)<br>BD Cat#555976 clone 12G5<br>mouse anti CD235a-APC (BD Cat#551336 clone HIR-2).
Validation	All antibodies used in zebrafish experiments were validated in the laboratory via performing immuno-fluorescence controls. All antibodies against human proteins were commercially validated.

Eukaryotic cell lines

Policy information about cell lines and Sex and Gender in Research

Cell line source(s)	WA01 (H1) were obtained from WiCell Stemcell bank.
Authentication	No new lines were generated, no lines were authenticated
Mycoplasma contamination	H1 line tested negative for mycoplasma contamination.
Commonly misidentified lines (See ICLAC register)	No commonly misidentified lines in the ICLAC registry are used in this study.

Animals and other research organisms

Policy information about studies involving animals; ARRIVE guidelines recommended for reporting animal research, and Sex and Gender in Research

Laboratory animals	Zebrafish animal were used in this study. Zebrafish adult (older than 3 months up to 1 year) were used to produce embryos. Here are all the transgenic lines used in this study that were previously characterized:<br>miR-128ya315-316 (ZDB-CRISPR-161031-5 and ZDB-CRISPR-161031-9)<br>Tg(kdrl:gfp)zn1 (ZDB-ALT-070529-1)<br>Tg(kdrl:hras-mCherry)s896 (ZDB-ALT-081212-4)<br>Tg(cmyb:GFP)zf169 (ZDB-ALT-071017-1)<br>Tg(7xTCF-Xla.Sia:NLS-mCherry)ia5 (ZDB-TGCONSTRCT-110113-2)<br>(Tg:TP1:eGFP)um14 (ZDB-ALT-090625-1)<br>Tg(runx1:eGFP) y509 (ZDB-360 ALT-170717-3,
Wild animals	The study doest not have wild animals.
Reporting on sex	Zebrafish animals below a month post fertilization do not have sex identification, while animals above 3 months post fertilization, sex were included as variable and experiments were conducted as 50% males and 50% females.
Field-collected samples	Zebrafish were raised and maintained at 28.5C using standard methods.

Ethics oversight	All Zebrafish experiments were approved by the Yale University Institutional Animal Care and Use Committee (#2017-11473).

Note that full information on the approval of the study protocol must also be provided in the manuscript.

Flow Cytometry

Plots

Confirm that:

☒ The axis labels state the marker and fluorochrome used (e.g. CD4-FITC).

☒ The axis scales are clearly visible. Include numbers along axes only for bottom left plot of group (a 'group' is an analysis of identical markers).

☒ All plots are contour plots with outliers or pseudocolor plots.

☒ A numerical value for number of cells or percentage (with statistics) is provided.

Methodology

Sample preparation	For adult Whole Kidney Marrow flow cytometry:<br>Adult WKM (1 month-old or 2 months-old) were mechanically dissociated as previously published. Quickly, after being anesthetized, ventral section was processed and whole kidney marrow was collected.<br>For head and trunk dissociation:<br>Fish at 26 hours post fertilization were anesthetized and trunk and head dissociation were processed through a diagonal cut at the basis of the yolk sac extension.<br>Dissected embryos were placed in PBS 1X (pH 7.4, Invitrogen) and were dissociated into single cell suspensions through treatment with liberase enzyme (Roche) for 1 hour at 28C. Liberase was then inactivated with fetal bovine serum (Thermofisher) and cell suspension were washed with cell suspension media (0.5% FBS, 0.8uM CaCl2, 1% Penicillin, leibovitz medium L15 380 (Gibco))73.DAPI was then used in cell suspensions to differentiate alive cells.<br><br>For human experiments:<br>Day 3 cells were trypsinized for 5 minutes and washed 5 times in IMDM, 10% FBS, and 10 µg/mL. Day 8 cells were trypsinized for 8 minutes and washed 2 times in IMDM, 10% FBS, and 10 µg/mL. They were further dissociated with Collagenase I for 30 minutes and washed an additional 2 times in Stem Pro 34. All samples were stained in Stem Pro 34 media.
Instrument	Flow cytometry was performed on BD LSR Fortessa and the BD Aria was used to FACS isolation.
Software	BD FACS Diva was used for data acquisition and FlowJo was used for analysis.
Cell population abundance	For zebrafish experiments:<br>100,000 total events were recorded per samples for WKM analysis.<br>200,000 to 500,000 GFP+ cells (endothelial cells) were sorted per day from trunk at 26 hpf and 400,000 to 1,000,000 GFP+ cells (endothelial cells) were sorted per day from head at 26 hpf per sorting day.<br><br>For human experiments:<br>500,000 - 1,000,000 of KDR+ cells and 10,000 - 50,000 CD34+ cells were isolated for each experiment.
Gating strategy	For zebrafish experiments:<br>Viable cells were sorted using FSC-A/SSC-A and doublets were removed using FSC-H/FSC-W and SSC-H/SSC-W.<br>For GFP, control cells (GFP-) were used to gates only GFP+ cells.<br><br>For human experiment:<br>Viable cells were gated using FSC-A/SSC-A and doublets were removed using FSC-H/FSC-W and SSC-H/SSC-W.<br>Autofluorescent cells are removed using the PerCP channel.

☐ Tick this box to confirm that a figure exemplifying the gating strategy is provided in the Supplementary Information.
