# Supplementary figures and images for: Haematopoietic stem and progenitor cell heterogeneity is inherited from the embryonic endothelium
Source: Nat Cell Biol. 2023 Jul 17;25(8):1135–45. doi: 10.1038/s41556-023-01187-9 (PMC10415179; doi:10.1038/s41556-023-01187-9)

# Gel images used in Extended Data Fig. 5h

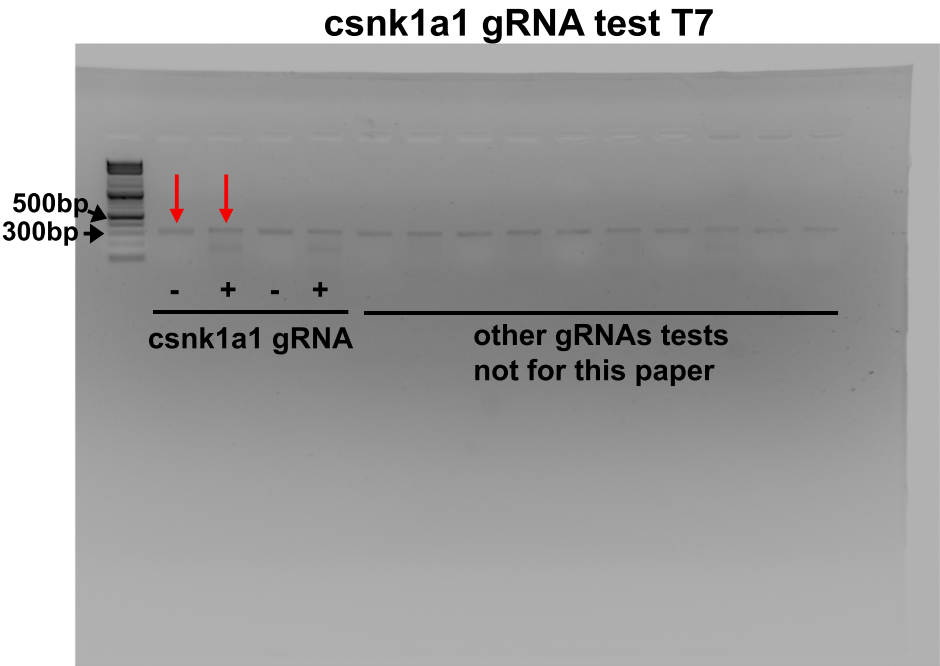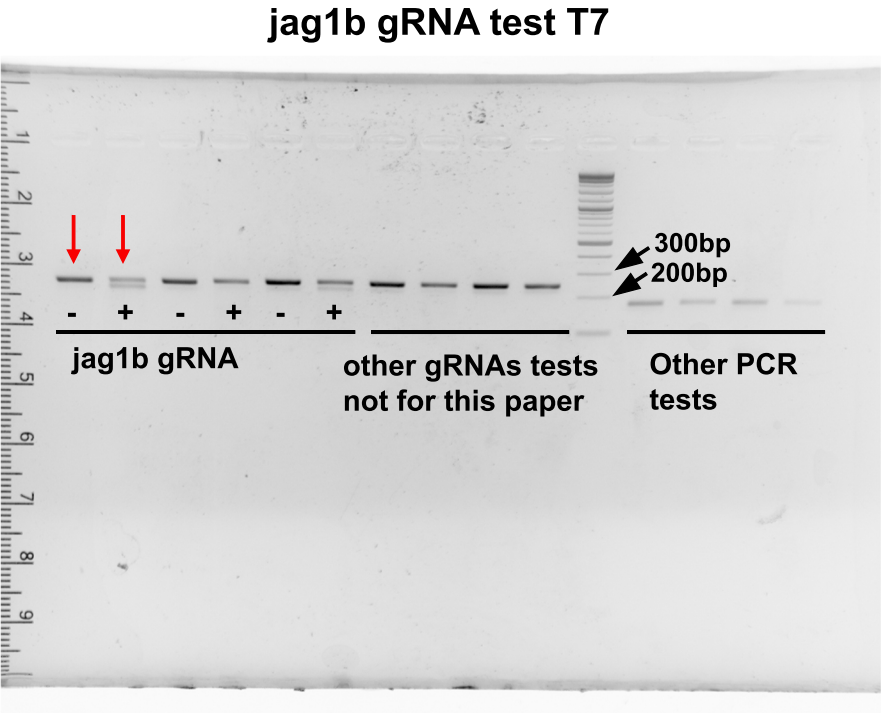

Supplement: Source Data Fig. 1 — Uncropped gel images used in Extended Data Fig. 5h. [file 41556_2023_1187_MOESM4_ESM.pdf]
